# PKA signaling modulates PRMT5/hnRNP A1-mediated IRES translation and dictates responses to mTOR inhibition in glioblastoma

**DOI:** 10.1007/s11060-026-05564-w

**Published:** 2026-06-05

**Authors:** Sunil Kumar, Angelica Benavides-Serrato, Jacquelyn T. Saunders, Robert N. Nishimura, Joseph F. Gera

**Affiliations:** 1 Department of Research & Development, Greater Los Angeles Veterans Affairs Healthcare System, Los Angeles, CA USA; 2 Department of Medicine, David Geffen School of Medicine at UCLA, Los Angeles, CA 90095 USA; 3 Department of Neurology, David Geffen School of Medicine at UCLA, Los Angeles, CA 90095 USA; 4 Jonnson Comprehensive Cancer Center, Los Angeles, CA 90024 USA; 5 Molecular Biology Institute, University of California, Los Angeles, CA USA; 6 Greater Los Angeles VA Healthcare System, 16111 Plummer Street (151), Building 1, Room C111A, Los Angeles, CA 91343 USA

**Keywords:** PKA, PRMT5, HnRNP A1, IRES activity, MTOR inhibition, Glioblastoma

## Abstract

**Introduction:**

Resistance to mTOR inhibitors in glioblastoma (GBM) is mediated in part by activation of internal ribosome entry site (IRES)-dependent translation, enabling continued synthesis of key cell cycle regulators. This salvage mechanism involves methylation-dependent activation of the IRES-*trans*-acting factor (ITAF) hnRNP A1 by PRMT5. However, the upstream regulators of this PRMT5/hnRNP A1 axis remain unclear.

**Methods:**

To identify upstream effectors, a yeast two-hybrid screen using PRMT5 revealed PKA-Cα as an interacting partner. This interaction was validated via coimmunoprecipitation and colocalization in GBM cells. Functional relevance was assessed using biochemical, genetic, and pharmacological approaches to modulate PKA activity and examine effects on PRMT5 phosphorylation, hnRNP A1 methylation, IRES activity, and mTOR inhibitor resistance.

**Results:**

PKA associated with PRMT5 and was activated in response to mTOR inhibition, correlating with increased cyclin D1 and c-myc mRNA IRES activity. PKA knockdown or inhibition attenuated IRES activation following mTOR inhibitor exposure. PRMT5 was phosphorylated at ser15 by PKA and this modification was required for PRMT5–hnRNP A1 interaction and downstream arginine methylation at residues R218/R225. A non-phosphorylatable PRMT5 S15A mutant failed to bind hnRNP A1, whereas a phosphomimetic S15E variant enhanced this association. PKA activation increased hnRNP A1 methylation, while combined inhibition of PKA and mTOR reduced GBM cell viability, suppressed IRES-mediated translation, and induced apoptosis both in vitro and in intracranial xenografts.

**Conclusions:**

These findings identify a PKA/PRMT5/hnRNP A1 signaling axis that promotes IRES-dependent translation and contributes to mTOR inhibitor resistance in GBM. Dual inhibition of PKA and mTOR may represent a promising therapeutic strategy.

**Graphical abstract:**

**Figure:**
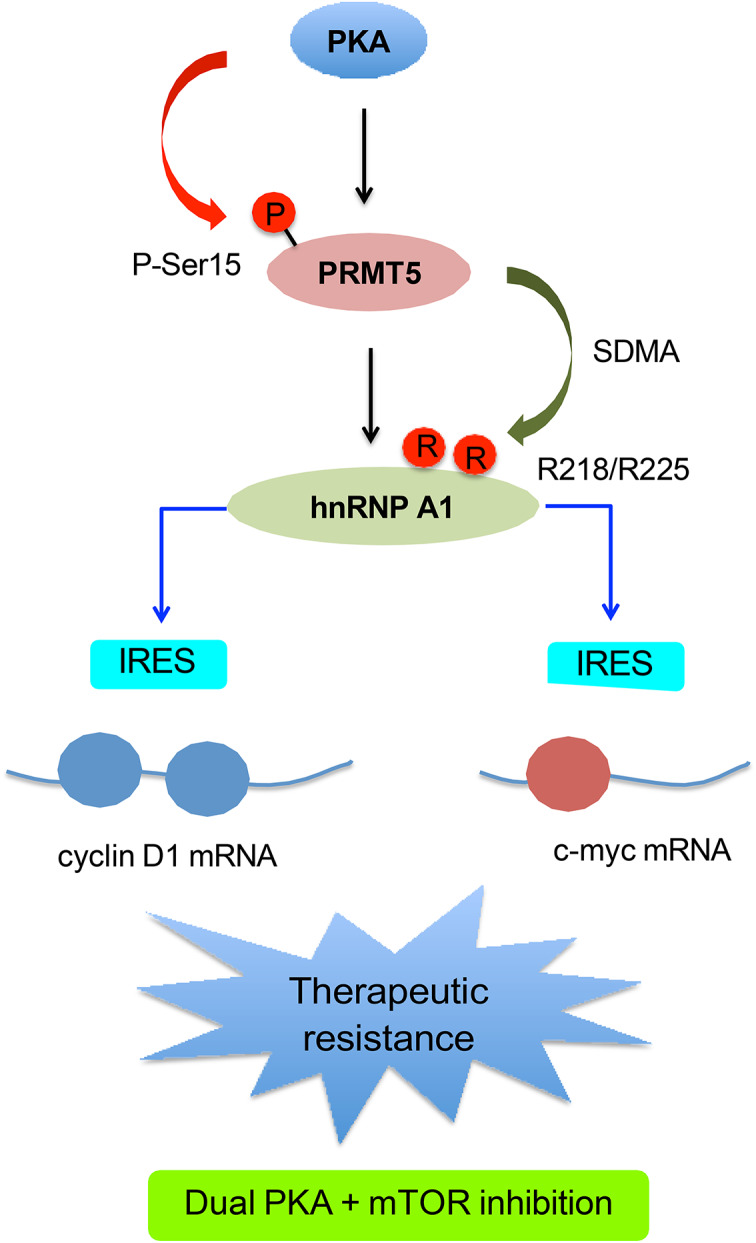
mTOR inhibition activates PKA in glioblastoma, leading to PRMT5 phosphorylation at Ser15 and enhanced interaction with hnRNP A1. This promotes PRMT5-dependent hnRNP A1 methylation and IRES-mediated translation of oncogenic proteins such as Cyclin D1 and c-MYC despite mTOR blockade. Dual inhibition of PKA and mTOR suppresses this adaptive translation program and overcomes therapeutic resistance

**Supplementary Information:**

The online version contains supplementary material available at 10.1007/s11060-026-05564-w.

## Introduction

Glioblastoma has a dismal median survival of only twelve months following diagnosis and is one of the most common central nervous system neoplasms [1, 2]. The lethality of this disease is due to the difficulties involved with complete surgical resection and the development of therapeutic drug resistance [3–5]. Common mutations in GBM driving tumor formation and progression include EGFR amplification events or the elevated expression of constitutively activating mutations of EGFR [6, 7]. Additionally, PTEN loss and phosphatidylinositol 3-kinase (PI3K) signaling hyperactivation are common. These tumor cell signaling drivers converge on the central downstream effector mTOR whose functions include regulating protein synthesis, metabolism, cell survival and drug resistance [8, 9]. The mTOR kinase associates with different regulatory components that define two active complexes (mTORC1 and mTORC2) each having distinct substrates [10–12].

Unfortunately, the success of mTOR inhibitors in the clinic for the treatment and management of GBM has been limited. This has been due to the loss of feedback regulation promoting AKT activation with first-generation allosteric inhibitors and toxicity issues associated with second-generation direct mTOR kinase inhibitors, although continuing clinical trials with direct mTOR kinase inhibitors, which inhibit both mTORC1 and mTORC2, await further evaluation [13–18]. Third-generation bivalent mTOR inhibitors, which have demonstrated remarkable preclinical activity, are currently in clinical trials [19–22]. Collectively these studies underscore the possible role of mTOR inhibitors as a treatment option for GBM.

The signaling interplay between mTOR complexes (mTORCs) indicates the existence of multiple potential mechanisms underlying resistance to mTOR-targeted therapies [23, 24]. Our findings reveal that both allosteric and ATP-competitive mTOR kinase inhibitors can induce a transcript-selective protein synthesis salvage pathway, which sustains the translation of crucial cell cycle regulatory mRNAs and thereby contributes to therapeutic resistance [25]. This compensatory translational response is mediated through internal ribosome entry site (IRES)-dependent mechanisms and requires the activity of the IRES *trans*-acting factor (ITAF), heterogeneous nuclear ribonucleoprotein A1 (hnRNP A1) [26].

Previous studies from our group identified the protein arginine methyltransferase PRMT5 as a critical mediator of hnRNP A1-dependent activation of cyclin D1 and c-myc IRES activity in GBM cells following treatment with mTOR inhibitors [26]. Specifically, PRMT5 was shown to symmetrically di-methylate arginine (SDMA) residues 218 and 225 on hnRNP A1, thereby enhancing its binding affinity to IRES RNA elements and promoting IRES-driven translation [26, 27]. In the present study, we expand upon these findings by identifying an upstream regulatory mechanism of the PRMT5/hnRNP A1/IRES axis. We demonstrate that PRMT5 serves as a direct substrate of PKA in vitro, and that phosphorylation of PRMT5 at serine 15 potentiates its methyltransferase activity toward hnRNP A1, correlating with increased cyclin D1 and c-myc IRES activity in GBM cells. Furthermore, combinatorial inhibition of PKA and mTOR signaling results in anti-tumor effects in both established and patient-derived GBM cell lines in vitro, as well as in an orthotopic xenograft mouse model.

## Materials and methods

Details regarding cell cultures, reagents, in vitro and in vivo protocols and data analyses are described in Online Resource 1 Supplemental Materials and Methods.

## Results

### PKA-Cα interacts with PRMT5

**Fig. 1:**
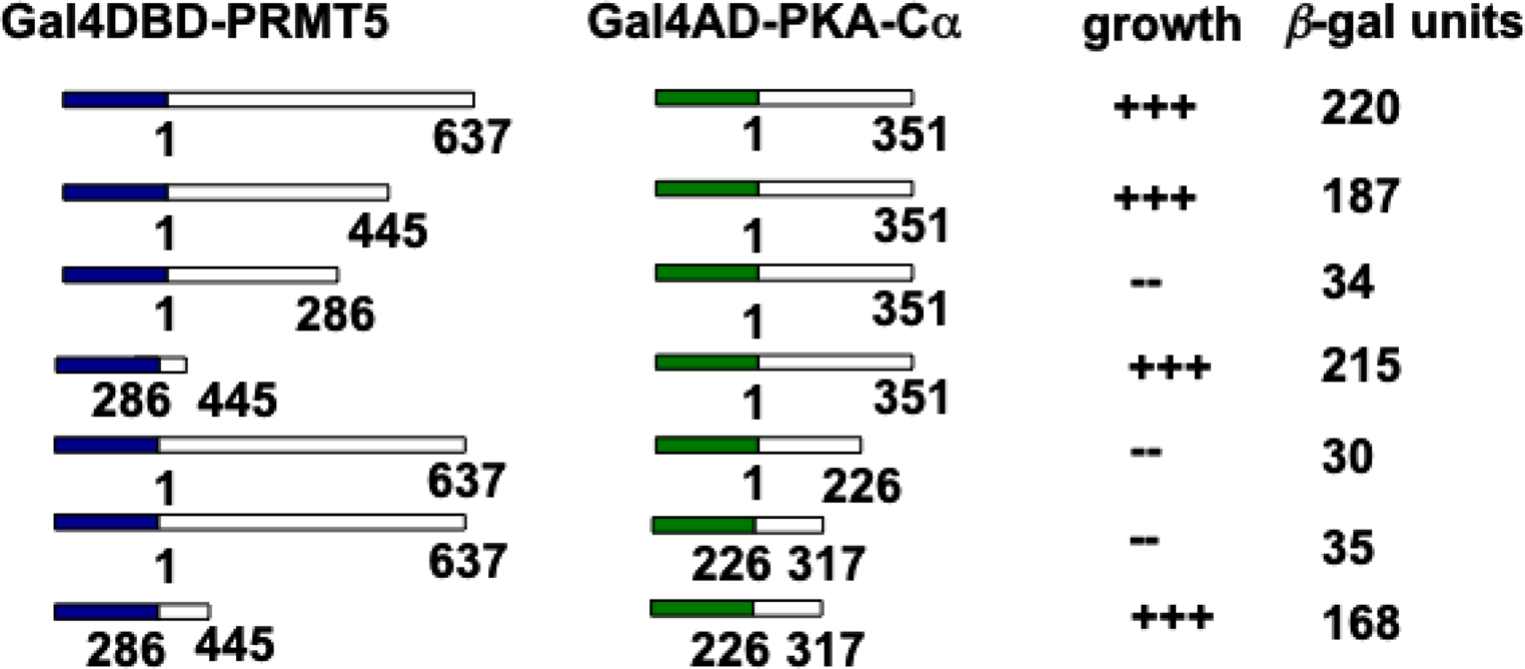
Mapping of interacting regions of PRMT5 and PKA-Caα via yeast two-hybrid assay. Full-length human PRMT5 (a.a. 1–637) was fused to the yeast Gal4 DNA-binding domain (DBD) and the full-length human PKA-Cα (a.a. 1–351) was fused to the yeast Gal4 activation domain (AD). The indicated deletion mutants were cotransfected into the AH109 two-hybrid strain and plated onto selective media to ascertain whether interaction between the proteins was detectable via transactivation of a HIS3 reporter. Growth is indicated by +++, corresponding to strong growth; ++, moderate growth; and -, no growth. Colonies which grew on selective media were also subjected to liquid *β*-gal assays to determine LacZ reporter activity

To begin to identify upstream effectors of the PRMT5/hnRNP A1 pathway we conducted high-throughput yeast two-hybrid screens utilizing full-length human PRMT5 as bait. We identified several known interactors of PRMT5 (Online Resource 2, Table 1) and additionally, the alpha catalytic subunit of PKA was identified in 7% of the total clones recovered. PKA activity has been previously linked to the control of IRES activity [28, 29], thus we focused our attention on characterizing the possible involvement of PKA as a regulator of PRMT5/hnRNP A1-mediated IRES activity. We subsequently determined which regions of PKA-Cα and PRMT5 were required for binding in the yeast two-hybrid assay. A set of deletion mutants were generated (Fig. 1) and mapping of the interacting regions revealed that a 91 residue domain of the C-terminal end of PKA-Cα and a larger 159 residue domain of PRMT5 encompassing the methyltransferase domain were required for the interaction under selective media conditions and yielding high levels of *β*-gal reporter activity. These data suggested that domains within the C-terminal end of PKA-Cα and within the methyltransferase domain of PRMT5 mediate the interaction.

**Table 1:** Genetic interactors of PRMT5 identified in yeast two-hybrid screens

gene	accession no.	subcellular location*	description
MEP50/WDR77	NM_001317062.2	pm, n, c	methylosome protein 50
SNRPD1	NM_006938.4	n, c	small nuclear ribonucleoprotein Sm D1
NOVA1	NM_002515.3	n, nuc	RNA-binding protein Nova-1
PABPC1	NM_002568.4	n, c	poly(A) binding protein
PKA-Cα (PRKACA)	NM_002730.4	pm, n, c, mito	PKA catalytic subunit alpha
RPS13	NM_001017.3	n, nuc, c	ribosomal protein S13
EIF5	NM_001969.5	c	eukaryotic translation initiation factor 5
hnRNP A1	NM_002136.4	n, c	heterologous nuclear ribonucleoprotein A1
KRAS	NM_033360.4	pm, c	GTPase KRas

pm, plasma membrane; n, nuclear; nuc, nucleoli; c, cytoplasmic; mito, mitochondrion

## PKA-Cα associates with PRMT5 in GBM lines

**Fig. 2:**
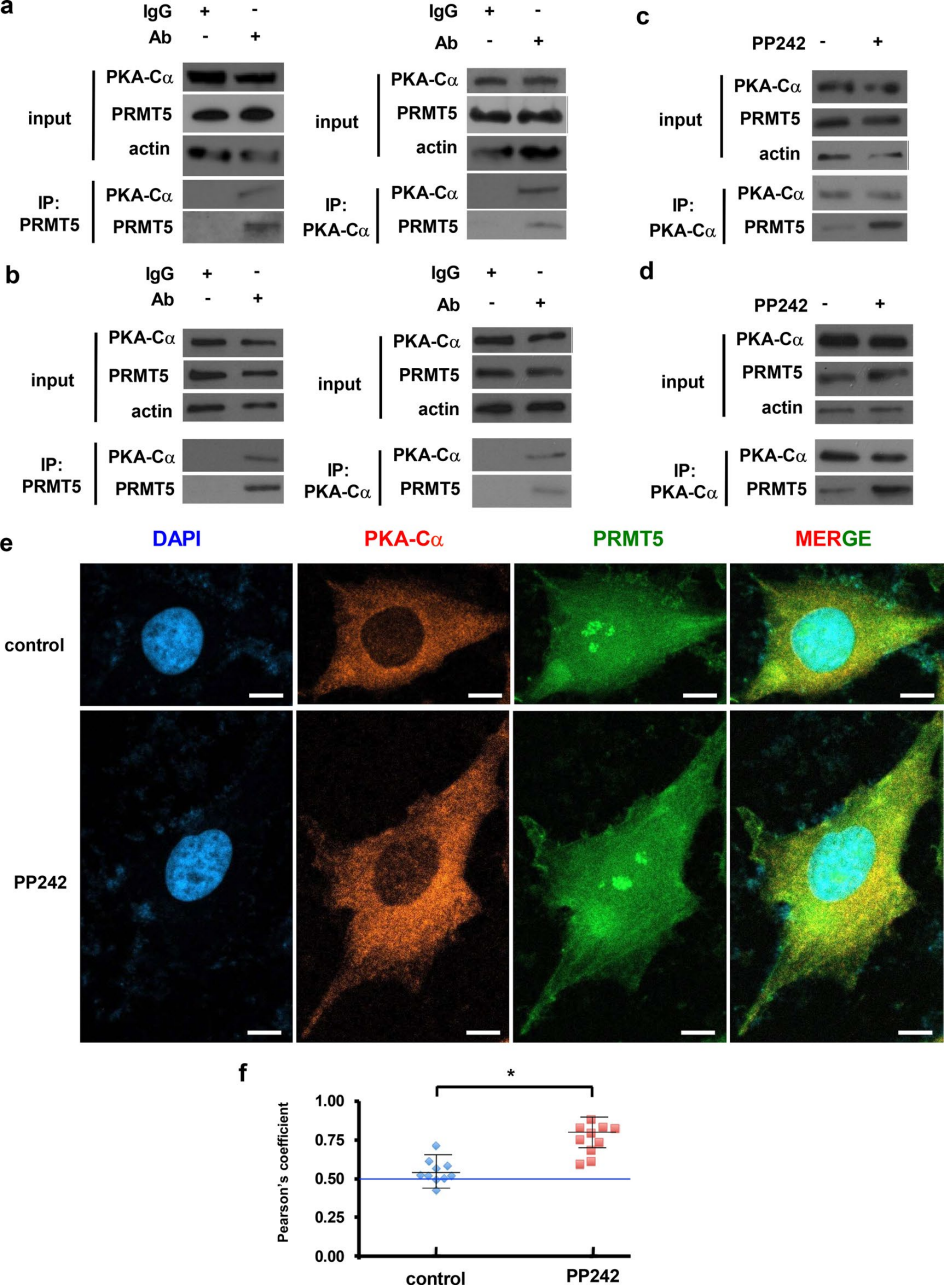
PKA-Cα interacts with PRMT5 in GBM cells. **a** Endogenous PRMT5 was immunoprecipitated from LN229 cells using α-PRMT5 antibodies coupled to Protein A-beads and immunoblotted for the indicated proteins (*left panel*). IgG, control serum. In the *right panel* endogenous PKA-Cα was immunoprecipitated from LN229 cells and probed for the indicated proteins, IgG, control serum. **b** as in **a**, except from GBM PDX HK296 cells. **c** endogenous PKA-Cα PRMT5 coimmunoprecipitation in the absence or presence of PP242 (100 nM, 4 h) in LN229 cells. **d** as in **c**, except from GBM PDX HK296 cells. **e** partial colocalization of endogenous PKA-Cα (red) and PRMT5 (green) in LN229 cells in media (control) and following treatment with PP242 (10 nM, 4 h). Scale bar, 10 µm. Panels on the right show merged composite images where the yellow color indicates areas of colocalization. **f** Pearson’s coefficient analysis of a representative experiment showing the means  ± S.D. obtained from single cells of ten fields. *, *p* < 0.05

To further confirm the direct binding of PKA-Cα with PRMT5 in GBM we performed coimmunoprecipitation experiments in LN229 and GBM PDX HK296 cells. As shown in Fig. 2a, endogenous PKA-Cα was detected in immunoprecipitates of PRMT5 (*left panel*) and in a reciprocal fashion, PRMT5 was found in immunoprecipitates of PKA-Cα (*right panel*). PKA-Cα and PRMT5 were observed to coimmunoprecipitate in a similar fashion from HK296 cells (Fig. 2b). Subsequently, we assessed the interaction dynamics of PKA-Cα and PRMT5 following mTOR inhibitor treatment. As shown in Fig. 2c, in LN229 cells, the association of PKA-Cα and PRMT5 was markedly increased in PKA-Cα immunoprecipitates following PP242 treatment compared to control. PP242 treatment also stimulated the binding of PRMT5 to PKA-Cα in coimmunoprecipitates from PDX HK296 cells (Fig. 2d). We also performed dual labeling immunofluorescence confocal microscopy experiments to assess whether PKA-Cα and PRMT5 were colocalized in LN229 cells under basal conditions and following mTOR inhibition. As shown in Fig. 2e, PKA-Cα was predominantly found diffusely in the cytoplasm, while PRMT5 stained diffusely throughout the cell, with localized regions of intense staining within the nucleus under both basal conditions and following exposure to PP242 (mTORC1/2 inhibitor) suggesting partial cytoplasmic colocalization of PKA-Cα and PRMT5 under both conditions. We performed Pearson’s coefficient analyses which revealed a high cytoplasmic colocalization rate of PKA-Cα and PRMT5 under basal (control) conditions and was further enhanced in PP242 treated cells (Fig. 2f). Collectively, these findings support an association between PKA-Cα and PRMT5 in GBM cells under basal conditions which is further stimulated following mTOR inhibition.

## PKA activity is induced following mTOR inhibition, is required for mTOR inhibitor-induced IRES activity, and stimulates PRMT5/hnRNP A1 signaling

**Fig. 3:**
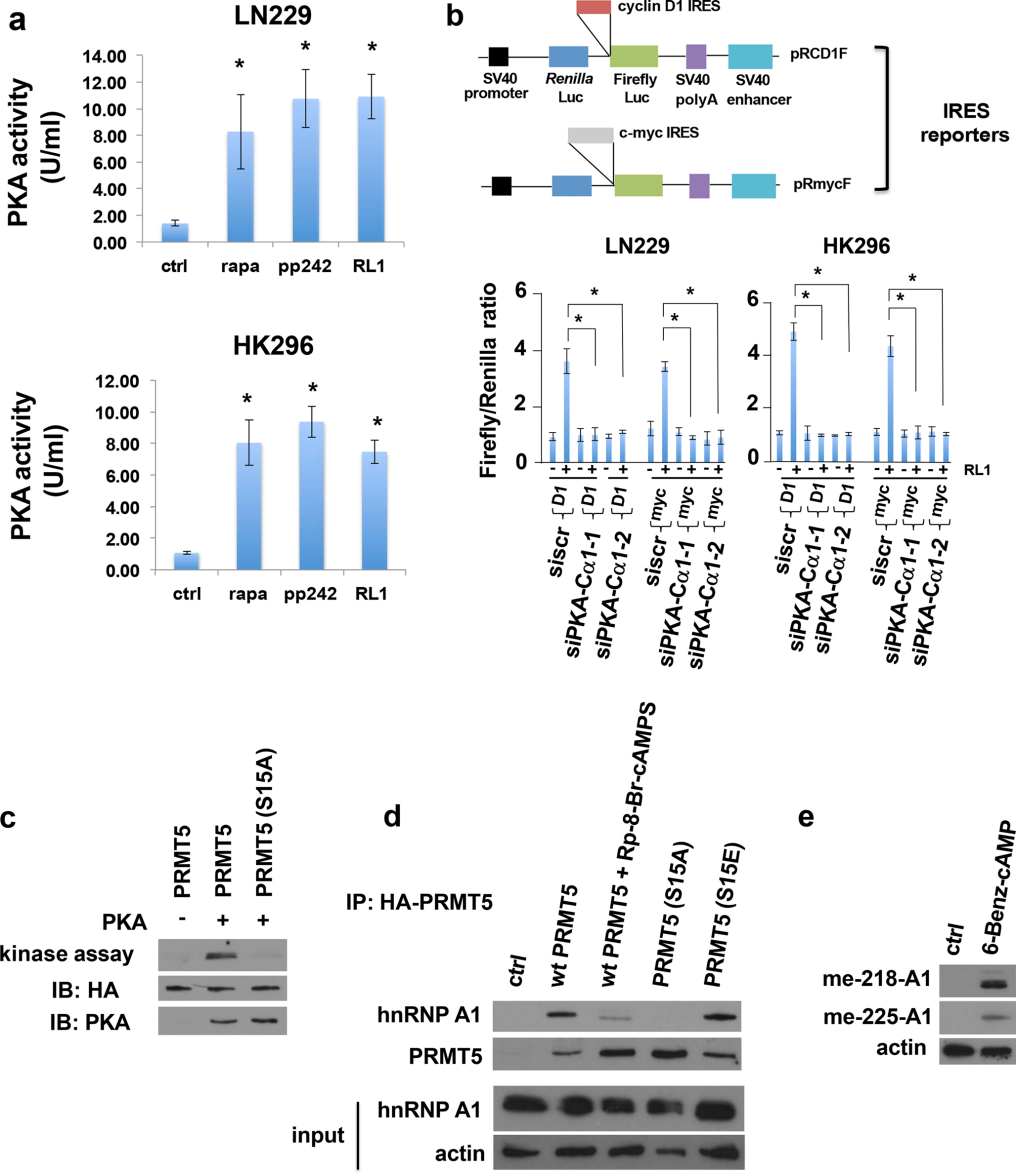
PKA activity is induced by mTOR inhibitors, is required for mTOR inhibitor-induced IRES activity, and phosphorylates PRMT5 leading to SDMA on hnRNP A1. **a** Established and patient-derived GBM lines were treated with the indicated mTOR inhibitors (100 nM for rapamycin and PP242, 1 nM for RL1 for 24 h) and lysates subjected to PKA activity assays. Mean  ± S.D. are shown, *n* = 3. *, *p* < 0.05 (rapamycin, PP242, RL1 versus control). **b** Cyclin D1 and c-myc RL1-induced IRES activity is blocked in GBM cells following PKA-Cα knockdown. Cells were transfected with PKA-Cα targeting siRNAs (1 & 2) or control scrambled siRNAs (siscr) followed by introduction of IRES reporters containing either the cyclin D1 or c-myc IRES sequences within the intercistronic regions (note firefly luciferase activity is a readout of IRES-dependent translation, whereas *renilla* luciferase activity corresponds to cap-dependent translation; see schematic diagrams, *top of figure*) and treated with RL1 (1 nM, 24 h) as indicated. IRES activity is expressed as the ratio of IRES-dependent firefly luciferase activity to cap-dependent *renilla* luciferase activity. Mean  ± S.D., *n* = 3. *, *p* < 0.05. **c** PKA phosphorylates PRMT5 on serine 15. LN229 cells were transfected with HA-tagged *wt* PRMT5 or a HA-tagged PRMT5 S15A nonphosphorylatable mutant and α-HA immunoprecipitate was incubated with active recombinant PKA-Cα and [γ-^32^P]ATP as shown. **d** PRMT5-knockout LN229 cells were transfected with the indicated HA-tagged PRMT5 alleles; *wt* PRMT5, *wt* PRMT5 + Rp-8-Br-cAMPS (PKA inhibitor, 100 µM, 4 h), nonphosphorylatable PRMT5 S15A or phosphomimetic PRMT5 S15E, treated with PP242 (100 nM, 4 h) and immunoprecipitated with α-HA antibodies. Immunoprecipitates were blotted for hnRNP A1 and PRMT5 as shown. **e** PKA activator 6-Benz-cAMP stimulates PRMT5-mediated SDMA 218 and 225 expression on hnRNP A1. LN229 cells were treated with the activator (25 µM, 4 h) and lysates probed using SDMA-specific antibodies for each arginine residue

We next determined what effect mTOR inhibition would have on PKA activity in GBM lines. As shown in Fig. 3a, treatment of LN229 or the PDX line HK296 with the mTOR inhibitors, rapamycin (mTORC1 inhibitor), PP242 (mTORC1/2 inhibitor), or Rapalink-1 (RL1) (3^rd^ generation bivalent mTORC1 inhibitor) resulted in a marked stimulation of PKA activity. We subsequently examined whether silencing PKA-Cα via two independent siRNAs (see Online Resource 3, Supplementary Fig. S1a) would block RL1-induced IRES activity. As shown in Fig. 3b, PKA-Cα knockdown effectively inhibited RL1-induced IRES activity in LN229 and HK296 cells. We subsequently explored whether PKA activity may regulate PRMT5. In a previous proteomics study [30] a major phosphorylation event at serine 15 of PRMT5 within the unique N-terminal triose phosphate isomerase (TIM) barrel was identified, however its potential as a PKA phosphosite was not investigated. To determine whether serine 15 could indeed be phosphorylated by PKA-Cα we performed in vitro kinase assays utilizing active recombinant PKA and purified recombinant native HA-tagged PRMT5, as well as a S15A substitution mutant. As shown in Fig. 3c, while native PRMT5 was efficiently phosphorylated by PKA-Cα, the S15A mutant did not display significant phosphorylation demonstrating that serine 15 could be phosphorylated by PKA-Cα in vitro. We then examined whether in LN229 cells in which PRMT5 had been knocked out via CRISPR-Cas9 editing (Online Resource 3, Supplementary Fig. S1b), reintroduction of various HA-tagged PRMT5 alleles affected the ITAF, hnRNP A1‘s ability to bind to PRMT5 following PP242 exposure. As shown in Fig. 3d, transfection of HA-tagged *wt* PRMT5 and subsequent immunoprecipitation using HA antibodies effectively coimmunoprecipitated hnRNP A1 with PRMT5, however when these cells were treated with the PKA inhibitor Rp-8-Br-cAMPS this significantly inhibited hnRNP A1 binding to PRMT5, as did cells transfected with the nonphosphorylatable PRMT5 S15A mutant. However, transfection of cells with a phosphomimetic PRMT5 S15E substitution mutant modestly enhanced hnRNP A1 binding relative to *wt* PRMT5. To determine whether PKA activation would lead to PRMT5-mediated SDMAs on hnRNP A1 we stimulated PKA with the cell permeable, PDE resistant, agonist 6-Benz-cAMP. As shown in Fig. 3e, expression of both SDMAs 218 and 225 on hnRNP A1 was induced following treatment in LN229 cells. These data support the notion that mTOR inhibition leads to activation of PKA and subsequent phosphorylation of serine 15 of PRMT5 resulting in increased binding and symmetric dimethylation of the ITAF, hnRNP A1 promoting IRES activity in GBM.

## Anti-glioblastoma effects of combined PKA and mTOR inhibition

**Fig. 4:**
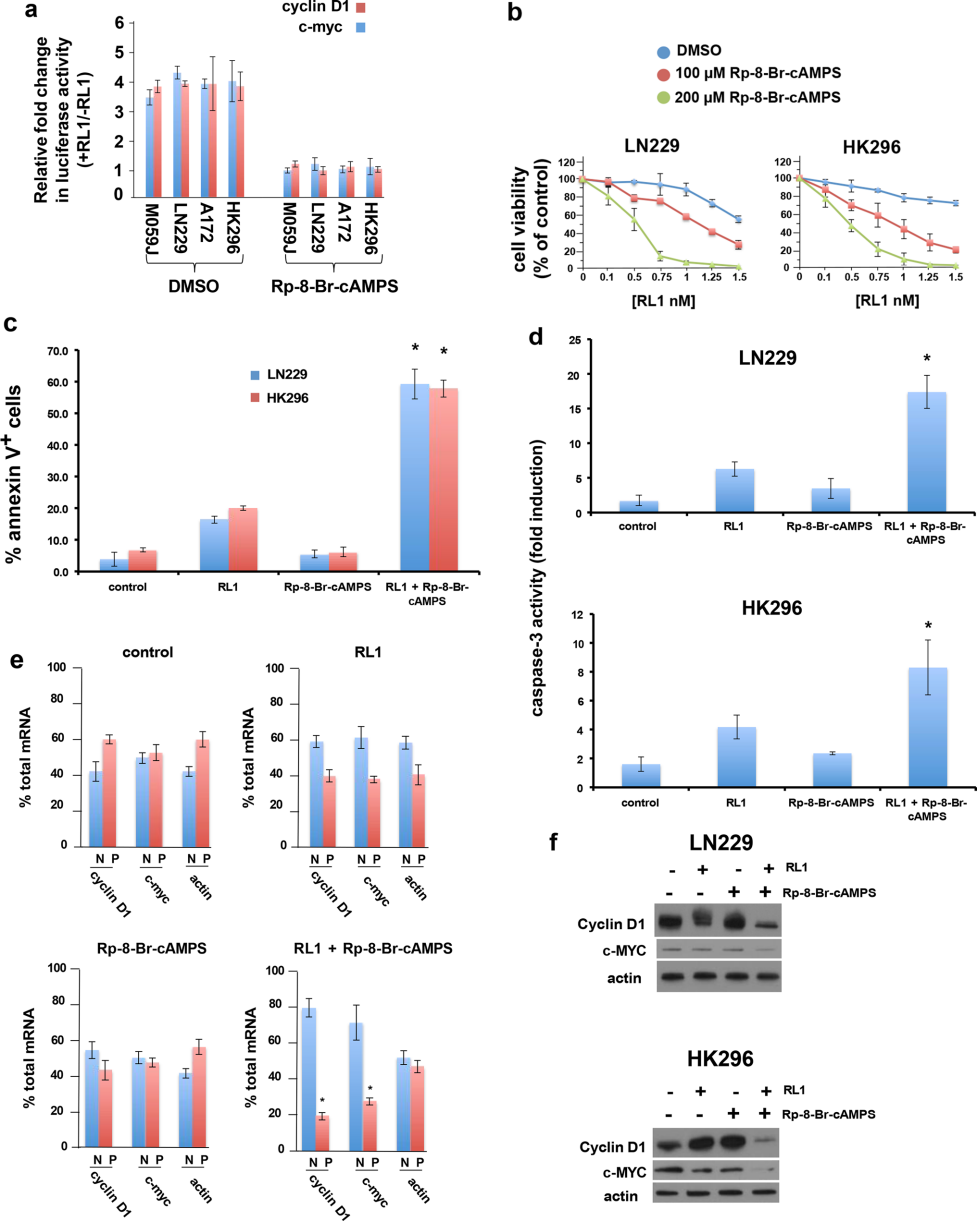
GBM cell responses to combined PKA and mTOR inhibition. **a** PKA inhibitor (Rp-8-Br-cAMPS) abrogates RL1-induced cyclin D1 and c-myc IRES activity in GBM lines. Cells were transfected with cyclin D1 or c-myc IRES reporters and treated with or without RL1 (1 nM, 24 h) and Rp-8-Br-cAMPS (200 µM, 24 h) as indicated. Firefly luciferase activity was subsequently determined in these cell extracts and expressed as the relative fold-change in activity. Mean  ± S.D., *n* = 3. **b** Rp-8-Br-cAMPS synergistically inhibits viability of LN229 and HK296 cells in cotreatments with RL1. Cells were treated with the indicated concentrations of RL1 alone or in combination with Rp-8-Br-cAMPS for 48 h and percent viability was determined as compared with control. Cell viability was assessed via ATP bioluminescence assays. Mean  ± S.D., *n* = 3. **c** the degree of apoptosis induction following cotreatments with RL1 and Rp- 8-Br-cAMPS was assessed via annexin V-FITC staining in LN229 and HK296 cells (RL1, 1 nM; Rp-8-Br-cAMPS, 200 µM; RL1 + Rp-8-Br-cAMPS, 1 nM and 200 µM, respectively at 48 h). Mean ± S.D., *n* = 3. *, *p* < 0.05 (RL1 +  Rp-8-Br-cAMPS versus Rp-8-Br-cAMPS, RL1, or control) **d** effects of combination RL1 and Rp-8-Br-cAMPS treatment on caspase-3 activity. Cells were treated as in **c**, and caspase-3 activity determined using Ac-DEVD-pNa as a substrate. Mean  ± S.D., *n* = 3. *, *p* < 0.05 (RL1 + Rp-8-Br-cAMPS versus Rp-8-Br-cAMPS, RL1, or control). **e** polysome analyses of HK296 cells treated with RL1 (1 nM, 24 h), Rp-8-Br-cAMPS (200 µM, 24 h), RL1 + Rp-8-Br-cAMPS (1 nM and 200 µM, respectively for 24 h) or control. RNAs were extracted and separated by sucrose gradient centrifugation and fractionated into polysomal (P) or non-ribosomal/monosomal (N) fractions. RNAs were then subjected to quantitative RT-PCR for the indicated transcripts and the mean  ± S.D., *n* = 3 are shown. *, *p* < 0.05 (polysomal CCND1 or c-myc; RL1 + Rp-8-Br-cAMPS treated versus polysomal CCND1 or c-myc in Rp-8-Br-cAMPS, RL1 or control). **f **Cyclin D1 and c-MYC protein levels following the indicated inhibitor treatments (RL1, 1 nM; Rp-8-Br-cAMPS, 200 µM; RL1 + Rp-8-Br-cAMPS, 1 nM and 200 µM, respectively) at 24 h after treatments in LN229 and HK296 cells

As RL1 has shown translational promise in preclinical studies we chose to focus on its activity relative to earlier mTOR inhibitors. In subsequent experiments we determined that the PKA inhibitor Rp-8-Br-cAMPS markedly inhibited RL1-induced cyclin D1 and c-myc IRES activity in several GBM lines (Fig. 4a), consistent with the PKA-Cα siRNA knockdown experiments (see Fig. 3b). To begin to determine if the combination of PKA and mTOR inhibition would have significant effects on GBM cell proliferation in vitro, we determined if GBM lines were susceptible to growth inhibition by Rp-8-Br-cAMPS. We did not observe significant effects on cell viability of these lines up to concentrations of 100 µM of Rp-8-Br-cAMPS (see Online Resource 3, Supplementary Fig. S1c). We then tested LN229 and HK296 cells at 100 and 200 µM Rp-8-Br-cAMPS concentrations in combination with a wide range of RL1 concentrations for their effects on cell viability. As shown in Fig. 4b, Rp-8-Br-cAMPS in combination with RL1 demonstrated remarkable anti-GBM effects on cell viability (LN229, CI = 0.24 at ED_50_ ratio of 1:100; HK296, CI = 0.17 at ED_50_ ratio of 1:100) (Fig. 4b). The combination treatment also markedly induced apoptosis in GBM lines as determined by annexin V staining (Fig. 4c) and caspase 3 activation (Fig. 4d). Rp-8-Br-cAMPS alone resulted in minimal induction of apoptosis while RL1 treatment alone displayed a significant induction of apoptosis under the conditions tested. However, the combination of both inhibitors led to a marked increase in apoptotic cells in these GBM lines. In a complementary approach, we also extended our studies to examine the effects of RL1 in conjunction with PKA-Cα knockdown on cell viability and apoptosis induction. As shown in Supplementary Fig. S2 (Online resource 4), treatment with RL1 in either LN229 or PDX HK296 cells in which PKA-Cα had been knocked down via two independent siRNAs resulted in marked reductions in cell viability (Fig. S2a,b) and induced apoptosis (Fig. S2c) as compared to controls. We additionally examined the mRNA translational state of the cyclin D1 and c-myc mRNAs in response to combination treatments in the PDX HK296 cells. Polysome analyses was performed and as shown in Fig. 4e, in RL1 treated cells cyclin D1 and c-myc mRNAs were redistributed to poorly translated fraction pools compared to controls (control values; cyclin D1, non-ribosomal/monosomal 41.2%; polysomal 58.8%; c-myc, non-ribosomal/monosomal 48.5%; polysomal 51.5%) with 59.8% of cyclin D1 transcripts associated with non-ribosomal/monosomal material and 40.2% associated with polysomes. Similarly, 61.9% of c-myc mRNAs were redistributed to non-ribosomal/monosomes while 38.1% was found associated with polysomes consistent with mTORC1 inhibition. In Rp-8-Br-cAMPs treated cells no significant alteration of cyclin D1 and c-myc mRNA non-ribosomal/polysomal distributions were observed as compared to controls, however in combination RL1 and Rp-8-Br-cAMPS treated cells a marked redistribution of cyclin and c-myc mRNAs was observed with 80.5% and 72.2% of these mRNAs, respectively associated with non-ribosomal/monosomal pools consistent with a marked reduction in translational state. This reduction in cyclin D1 and c-myc mRNA translational efficiency was also supported by the observed protein levels in LN229 or HK296 cells treated with RL1 or Rp-8-Br-cAMPS alone or in combination as shown in Fig. 4f.

## Combination therapy of Rp-8-Br-cAMPS and RL1 in intracranial GBM xenografts

**Fig. 5:**
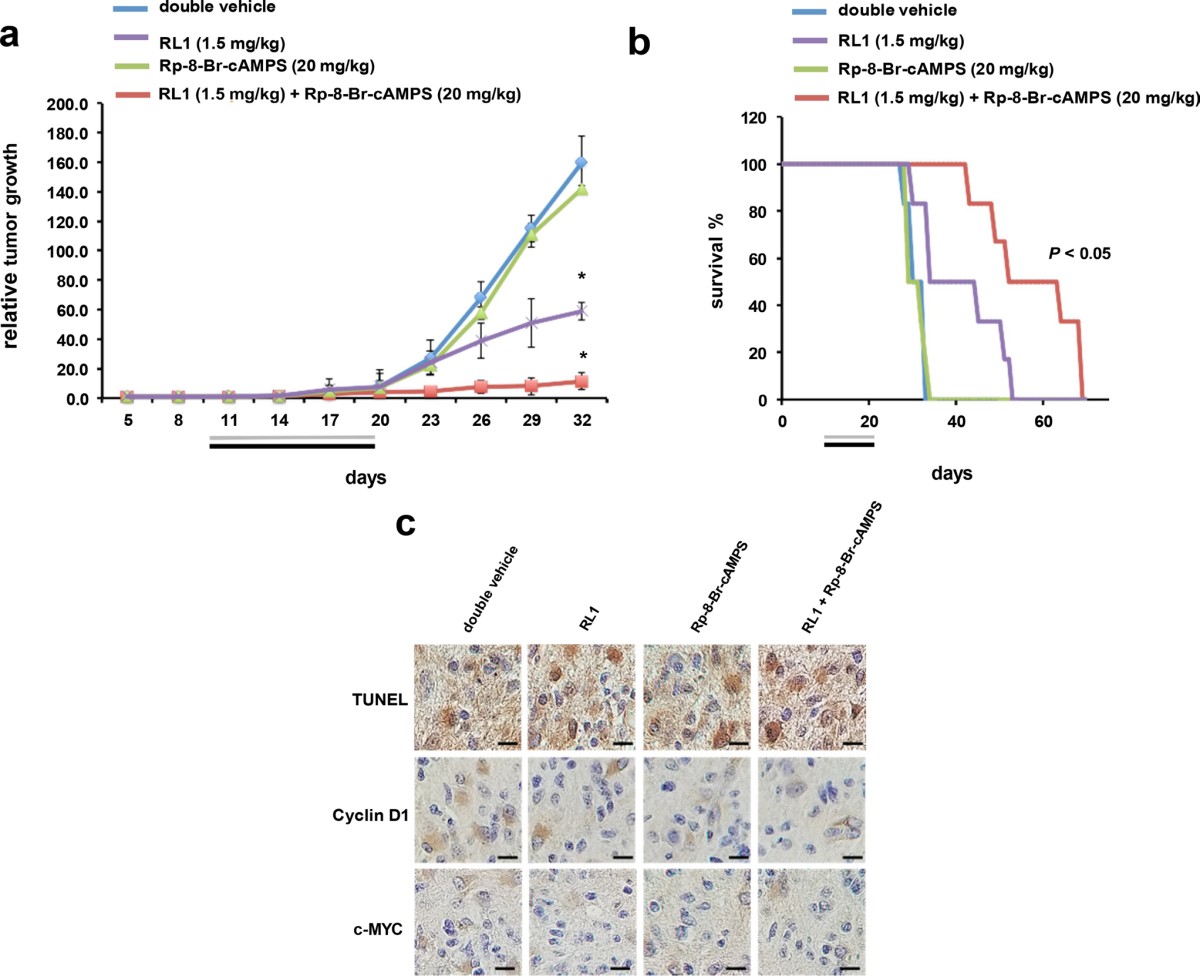

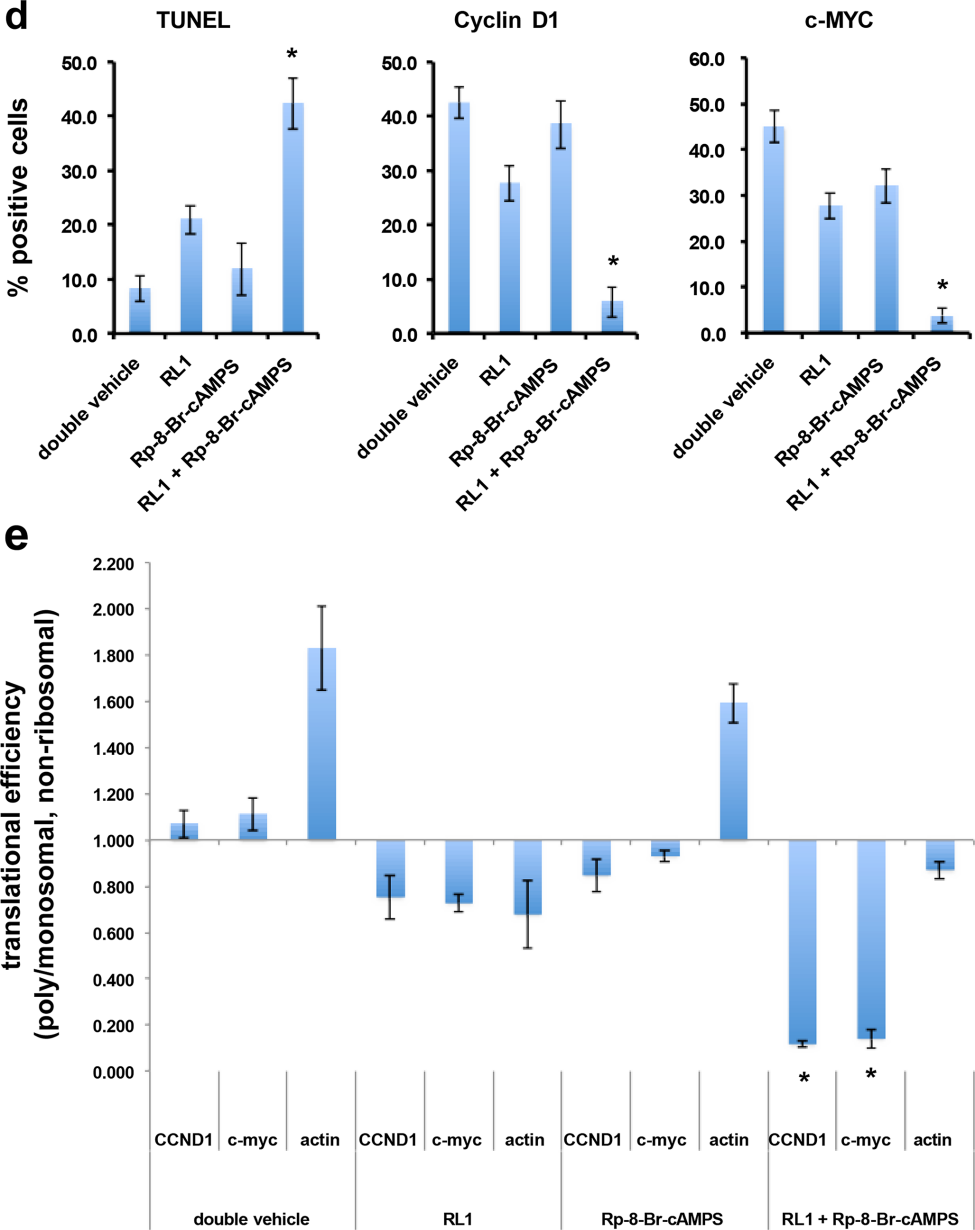
Co-treatment effects of RL1 and Rp-8-Br-cAMPS on GBM growth in vivo. **a** LN229 tumor burden of intracranial xenografted nude mice treated with double vehicle, RL1 alone, Rp-8-Br-cAMPS alone and the combination of RL1 + Rp-8-Br-cAMPS as shown. Treatment of mice began at day 10 post-implantation with Rp-8-Br-cAMPS administered intraperitoneally daily for 10 consecutive days at 20 mg/kg/d. RL1 (1.5 mg/kg) was administered intraperitoneally beginning on day 10, once every 5 days through day 20 post-implantation. Gray and black bars indicate treatment durations. Engrafted cells expressed firefly luciferase and bioluminescence intensity was determined over time. *, *p* < 0.05, *n* = 6 mice per group (RL1 + Rp-8-Br-cAMPS versus RL1 alone, Rp-8-Br-cAMPS alone, or double vehicle; RL1 alone versus Rp-8-Br-cAMPS alone or double vehicle. **b** survival curves of intracranially xenografted mice with LN229 cells. Beginning 10 days post-implantation mice were treated with the indicated inhibitors and dosing schedules as in **a**. *, *p* < 0.05, *n* = 6 mice per group, (RL1 + Rp-8-Br-cAMPS versus RL1 alone, Rp-8-Br-cAMPS alone, or double vehicle). **c** Representative images of harvested xenograft tumor sections (day 32) stained for TUNEL, Cyclin D1 and c-MYC from mice receiving the indicated treatments as shown. Scale bar, 20 µm. **d** Quantification of TUNEL, Cyclin D1 and c-MYC positive cells. Data are expressed as the number of positive cells (brown cells) divided by total cells per high power field (10–12 hpf/tumor; 3 tumors evaluated per group) values are means  ± S.D. *, *p* < 0.05. **e** histogram showing the translational efficiency of CCND1, c-myc or actin mRNAs from day 32 tumors as determined by quantitative RT-PCR as the ratio between polysomal and non-ribosomal/monosomal RNA pools. Mean and  ± S.D. are shown. (*, *p* < 0.05, *n* = 3, CCND1 or c-myc in RL1 + Rp-8-Br-cAMPS versus CCND1 or c-myc in RL1 alone, Rp-8-Br-cAMPS alone, or double vehicle)

To evaluate whether co-therapy of PKA and mTOR inhibition would lead to inhibition of in vivo tumor growth, we conducted intracranial xenograft studies in mice. Animals harboring intracranial xenografts of luciferase-tagged LN229 cells were randomized into treatment groups receiving double vehicle, RL1 (1.5 mg/kg every 5 days), Rp-8-Br-cAMPS (20 mg/kg/d), and RL1 (1.5 mg/kg every 5 days) + Rp-8-Br-cAMPS (20 mg/kg/d). As shown in Fig. 5a, xenografts receiving monotherapy with RL1 resulted in a significant reduction in tumor growth rate (69% inhibition at end of study). Tumor growth following treatment with Rp-8-Br-cAMPS did not differ from growth rates of double vehicle and was consistent with the lack of effects of this inhibitor by itself in vitro. However, the combination of RL1 and Rp-8-Br-cAMPS was significantly more effective then either of the monotherapies (94% inhibition at end of study). In agreement with the effects of cotherapy on xenograft growth, overall survival of animals was markedly extended in the group receiving combination PKA and mTOR therapy (Fig. 5b). Additionally, mice tolerated the dosing regimen without any obvious acute or chronic toxicities or weight loss. Moreover, apoptosis was significantly elevated in RL1 + Rp-8-Br-cAMPS harvested tumors as determined by TUNEL staining (Fig. 5c and d), relative to tumors from animals receiving either monotherapy alone. Cyclin D1 and c-MYC positive cells were also significantly reduced in tumors harvested from cotreated animals compared to those in monotreatment groups. PKA activity was also stimulated in harvested tumors from animals treated with RL1 consistent with our in vitro findings (Fig. 3a and Online Resource 3, Supplementary Fig. S1d). To support that the reductions in Cyclin D1 and c-MYC positive staining cells were due to effects on mRNA translation we examined the translational state of these transcripts by polysome analyses from these tumors. As shown in Fig. 5e, cyclin D1 and c-myc exhibited mRNA translational states of 1.06 and 1.11 (ratio of polysomal to nonribosomal/monosomal RNAs), respectively in tumors from mice administered double vehicle. Mice administered RL1 displayed reduced translational state ratios of 0.75 and 0.72 for cyclin D1 and c-myc mRNAs, respectively. Actin mRNA polysomal association was also reduced as it is translated via eIF-4E-dependent initiation indicating that RL1 treatment effectively inhibited mTORC1 mediated protein synthesis. Consistent with the in vitro polysome analyses results (see Fig. 4e), Rp-8-Br-cAMPS treatment alone also modestly reduced the translational state of the cyclin D1 and c-myc transcripts via effects on IRES-mediated translation while actin mRNA translational efficiency was not affected. Cyclin D1 and c-myc mRNAs from tumors of animals administered combination therapy showed a much greater reduction in translational efficiency consistent with the inhibition of both eIF-4E and IRES-dependent initiation.

## Discussion

In the present report we have extended our previous study in which we characterized a signaling pathway involving PRMT5 induced methylation of the ITAF, hnRNP A1 and subsequent activation of cyclin D1 and c-myc IRES-mediated protein synthesis leading to mTOR inhibitor resistance in GBM [26]. Here we identify PKA as an upstream effector of PRMT5/hnRNP A1/IRES signaling and demonstrate that mTOR inhibitors activate PKA activity and that PKA is required for mTOR inhibitor-induced IRES activity in GBM cells. We further show that serine 15 is a phosphorylation site for PKA on PRMT5 and that this phosphorylation event is required for hnRNP A1 binding and PRMT5-mediated methylation of symmetrical dimethylation events on hnRNP A1 upon PKA activation. Combination treatments utilizing Rp-8-Br-cAMPS and RL1 to inhibit PKA and mTORC1 activity, respectively demonstrated significant anti-GBM effects both in vitro and in intracranial xenografts. These data support PKA as an upstream effector of PRMT5/hnRNP A1/IRES signaling in GBM leading to mTOR inhibitor resistance.

Our domain mapping experiments identified the interaction between the C-terminal region of PKA-Cα and the methyltransferase domain of PRMT5, indicating that this interface is critical for complex formation. Disruption of these regions would likely reduce phosphorylation at PRMT5 Ser15, despite its location in the N-terminal region. Binding within the catalytic domain may allosterically modulate PRMT5 conformation, enhancing accessibility of the N-terminus or promoting productive kinase–substrate engagement. It is possible that physical association and phosphorylation are mechanistically coupled through long-range allosteric regulation for these protein partners, even across spatially distinct regions.

PKA is known to regulate the protein synthesis of several mRNAs which contain IRESs [28, 31]. Moreover, PKA has been shown to regulate several ITAFs directly such as PTB [29]. Additionally, it has been suggested that the IRES within the insulin receptor mRNA may be regulated by PKA activity such that glucose-induced PKA-mediated modification of PTB may contribute to enhanced IRES activity [28]. Our data support the notion that both the cyclin D1 and c-myc IRESs are regulated by PKA activity under conditions of mTOR inhibition when global cap-dependent protein synthesis is abrogated in GBM cells. The mechanism(s) by which PKA activity is induced in response to mTOR inhibitors is currently under investigation.

PKA activity has been linked to PRMT5 association during stimulation of CREB-target genes during pancreatic gluconeogenesis [32]. It was demonstrated that CRTC2, a transcriptional co-activator of CREB, associates with PRMT5, and enhanced PKA-mediated phosphorylation of CREB in response to glucagon [33]. PRMT5 was shown to potentiate CREB phosphorylation through increases in histone H3 Arg2 methylation which stimulated nucleosome clearance and accessibility of PKA. Our studies demonstrate a direct link between PKA and PRMT5 via phosphorylation at serine 15 (Fig. 3c& d). We also show that stimulation of PKA activity via the agonist 6-Benz-cAMP led to marked increases in PRMT5-mediated symmetrical dimethylation of arginines 218 and 225 on the ITAF hnRNP A1 (Fig. 3e). These methylation events lead to the activation of cyclin D1 and c-Myc IRES activity [26]. It has also been shown that PKC is able to phosphorylate PRMT5 on serine 15 to enhance p65 association and methyltransferase activity in response to IL-1β [30]. mTORC2 is known to phosphorylate and activate PKC [34, 35], thus blocking mTORC2 with mTOR kinase inhibitors or chronic rapamycin exposure would lead to decreased PKC activity and protein levels favoring PKA-mediated serine 15 PRMT5 phosphorylation in the context of mTOR inhibition.

The role of PKA activity in GBM remains complex and not fully understood [36]. Some studies suggest that PKA may act as a tumor suppressor [37, 38], while others indicate that its activation can promote tumor growth and invasion through downstream signaling of PDGFR in GBM [39]. PKA activity has also been implicated in contributing to drug resistance. For example, PKA activation has been associated with increased phosphorylation and activity of GSTP1, which may lead to treatment failure [40]. Furthermore, activation of the PKA/Dock180/Rac1 pathway has been shown to enhance GBM cell growth, motility, and invasiveness, potentially conferring resistance to therapy [39]. Our findings further support a role for PKA in promoting IRES-dependent translation, contributing to resistance to mTOR inhibition.

Although the precise mechanism by which mTOR inhibition leads to PKA activation remains to be fully defined, several plausible and non–mutually exclusive mechanisms have been proposed. mTOR inhibition is well known to relieve negative feedback loops within the PI3K/AKT signaling axis, resulting in compensatory pathway rewiring and enhanced upstream signaling [41, 42]. Such feedback relief can influence GPCR or receptor tyrosine kinase activity and potentially increase adenylyl cyclase–mediated cAMP production, thereby activating PKA. In addition, mTOR plays a central role in metabolic regulation, and its inhibition induces broad metabolic reprogramming that may alter intracellular ATP and cAMP dynamics or modulate phosphodiesterase activity, ultimately impacting PKA signaling [43]. Finally, mTOR suppression can trigger adaptive stress responses that engage compensatory survival pathways, including cAMP–PKA signaling, as part of a broader homeostatic network response [44].

A limitation of our current study is that orthotopic GBM PDX models were not evaluated in the context of combination PKA and mTOR inhibition. This is important, as established cell lines such as LN229 undergo extensive in vitro passaging, leading to clonal selection and reduced heterogeneity, thereby limiting their ability to recapitulate primary tumor biology. In contrast, orthotopic GBM PDX models preserve tumor heterogeneity and microenvironmental context, supporting the need for future in vivo studies to more accurately assess therapeutic efficacy.

Our work supports a model in which PKA is an upstream effector of the PRMT5/hnRNP A1 axis resulting in enhanced cyclin D1 and c-myc IRES-mediated protein synthesis and mTOR inhibitor resistance in GBM. PKA activity was demonstrated to be necessary for mTOR inhibitor-induced IRES activity and pharmacological inhibition of PKA resulted in synergistic anti-GBM responses together with mTOR inhibitors. Targeting PKA activity in combination with mTOR inhibition may find clinical utility.

## Electronic supplementary material

Below is the link to the electronic supplementary material.

Supplementary material 1

Supplementary material 2

## Data Availability

The datasets generated during the course of this study are available from the corresponding author upon reasonable request.
