# Supplementary material for: PKA signaling modulates PRMT5/hnRNP A1-mediated IRES translation and dictates responses to mTOR inhibition in glioblastoma
Source: J Neurooncol. 2026 Jun 5;178(2):45. doi: 10.1007/s11060-026-05564-w (PMC13241469; doi:10.1007/s11060-026-05564-w)

Supplementary data, uncropped immunoblots. Portions of the blots used to generate figures and supplementary figures are indicated by boxes.

Fig. 2a. *Left panel*, red boxes; *right panel*, blue boxes.

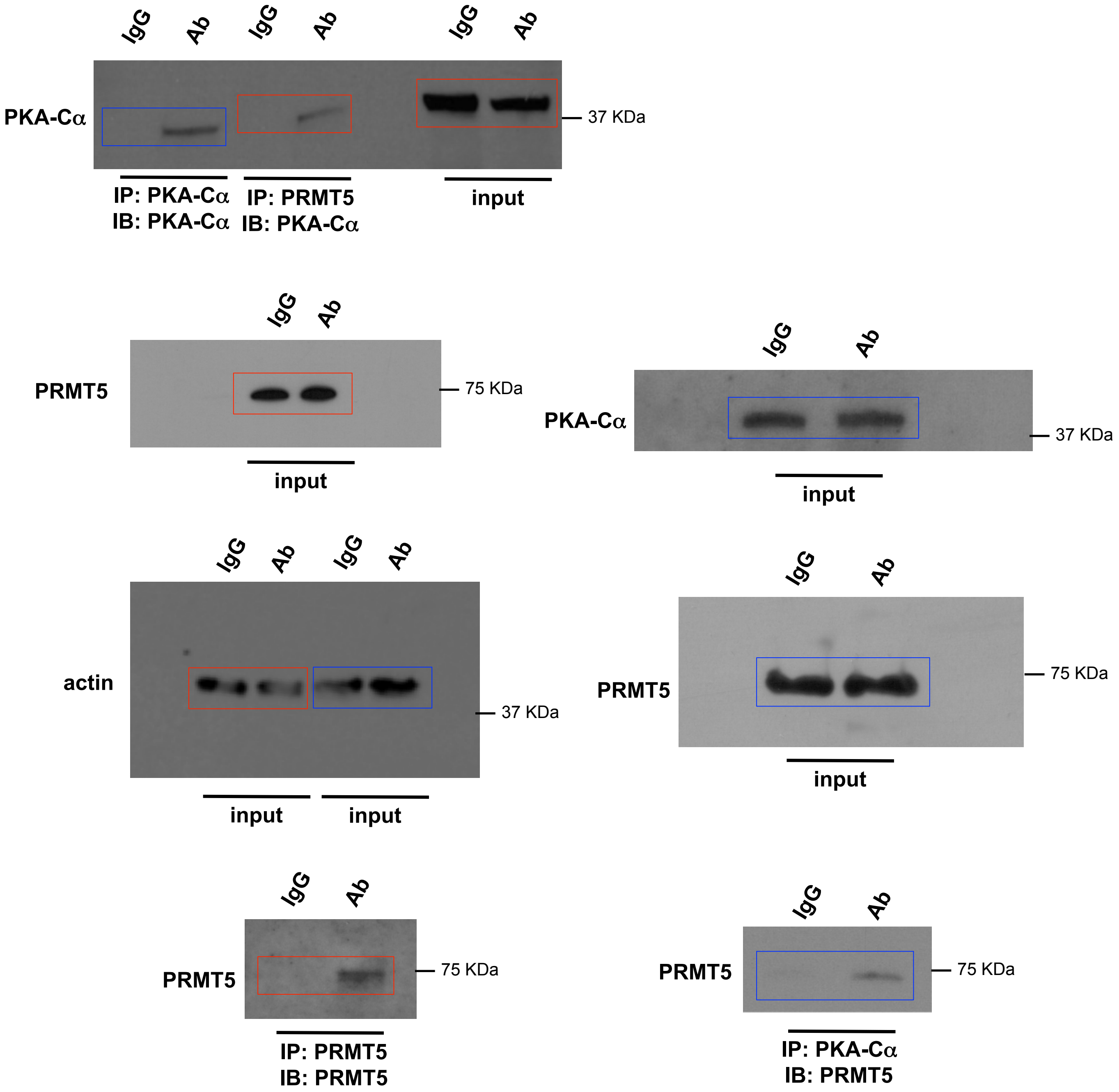

Fig. 2b. *Left panel*, red boxes; *right panel*, blue boxes.

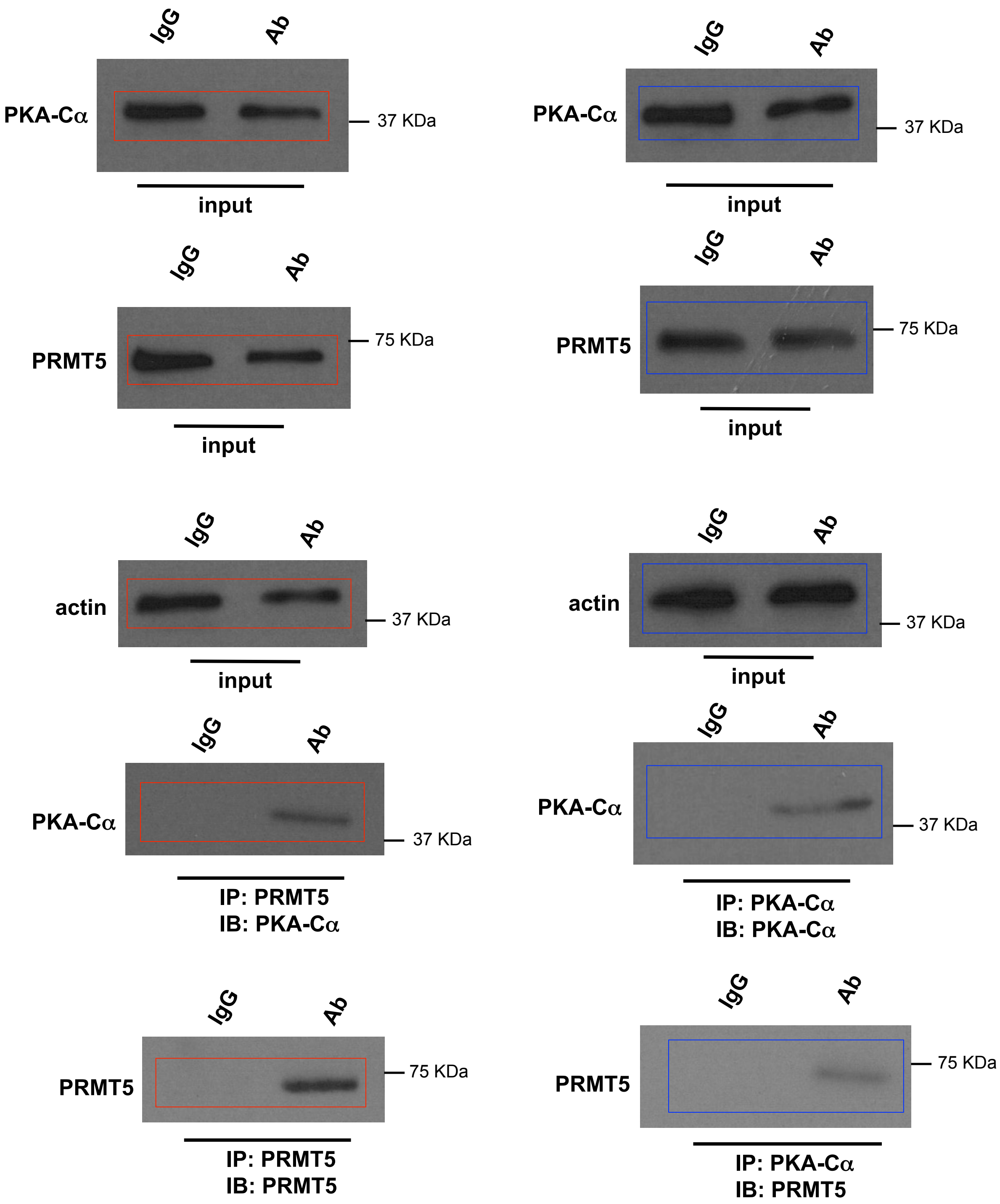

Fig. 2c.

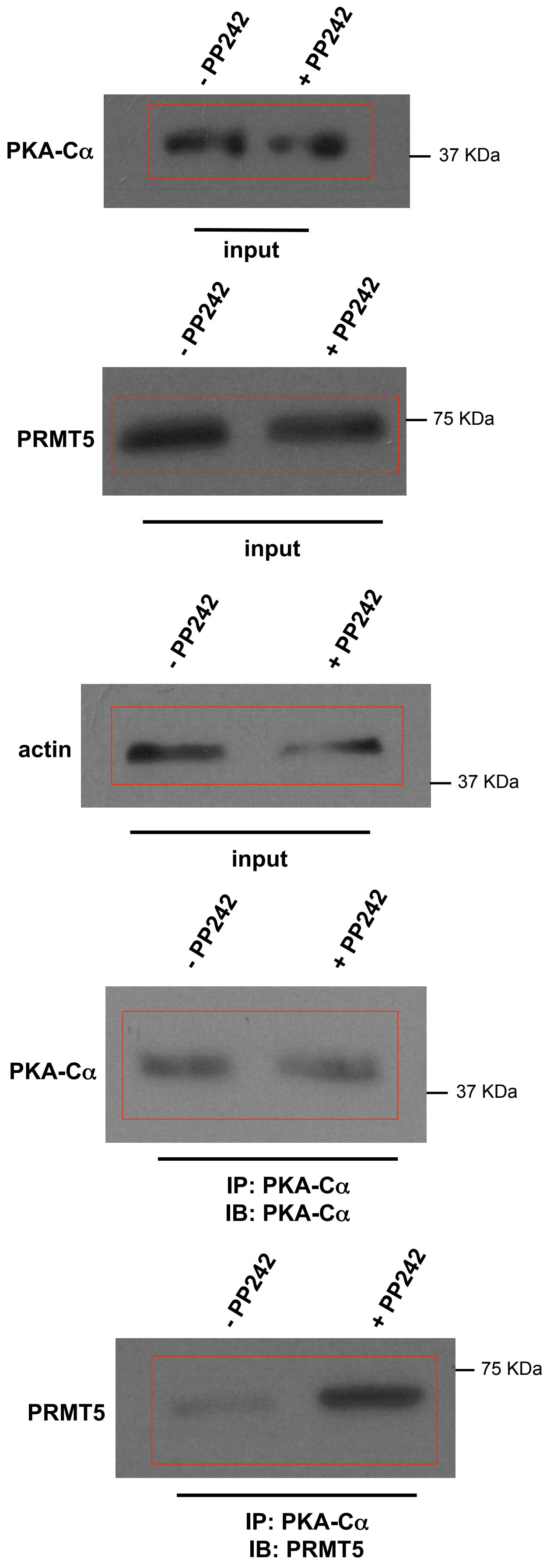

- PP242
+ PP242
PKA-Cα
37 KDa
input
- PP242
+ PP242
PRMT5
75 KDa
input
- PP242
+ PP242
actin
37 KDa
input
- PP242
+ PP242
PKA-Cα
37 KDa
IP: PKA-Cα
IB: PKA-Cα
- PP242
+ PP242
PRMT5
75 KDa
IP: PKA-Cα
IB: PRMT5

Fig. 2d.

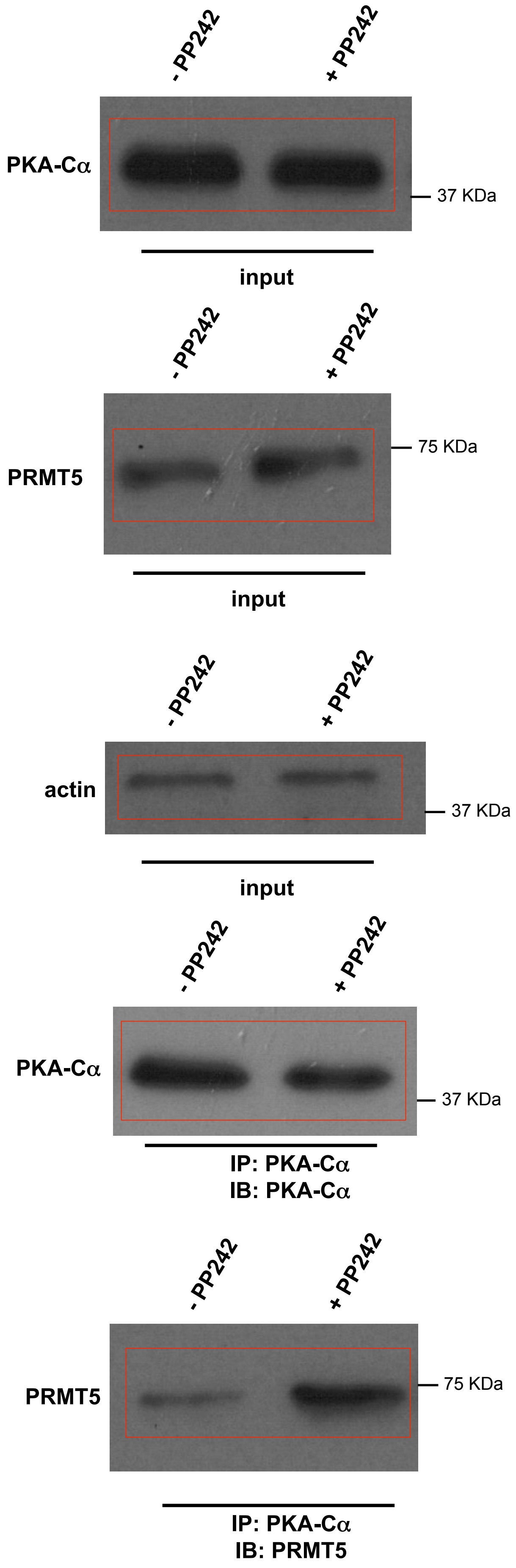
- PP242
+ PP242
PKA-Cα
37 KDa
input
- PP242
+ PP242
PRMT5
75 KDa
input
- PP242
+ PP242
actin
37 KDa
input
- PP242
+ PP242
PKA-Cα
37 KDa
IP: PKA-Cα
IB: PKA-Cα
- PP242
+ PP242
PRMT5
75 KDa
IP: PKA-Cα
IB: PRMT5

Fig. 3c.

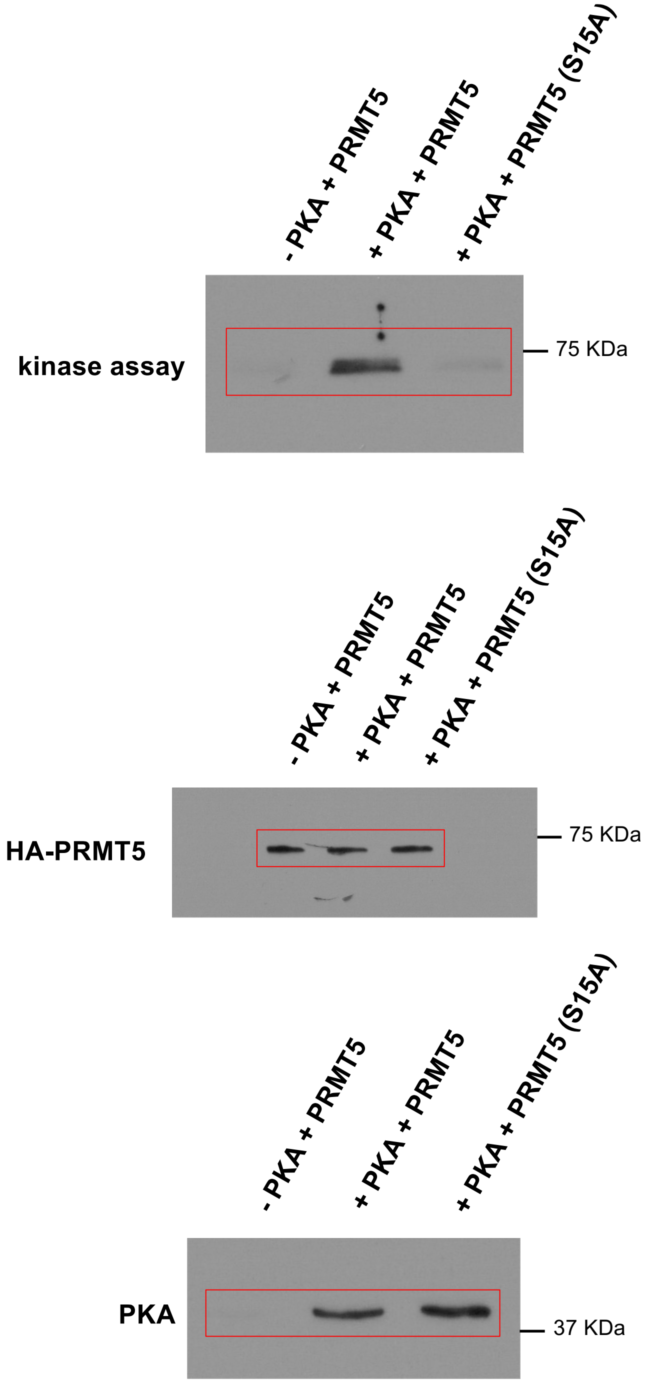
- PKA + PRMT5
+ PKA + PRMT5
+ PKA + PRMT5 (S15A)
kinase assay
75 KDa
- PKA + PRMT5
+ PKA + PRMT5
+ PKA + PRMT5 (S15A)
HA-PRMT5
75 KDa
- PKA + PRMT5
+ PKA + PRMT5
+ PKA + PRMT5 (S15A)
PKA
37 KDa

Fig. 3d.

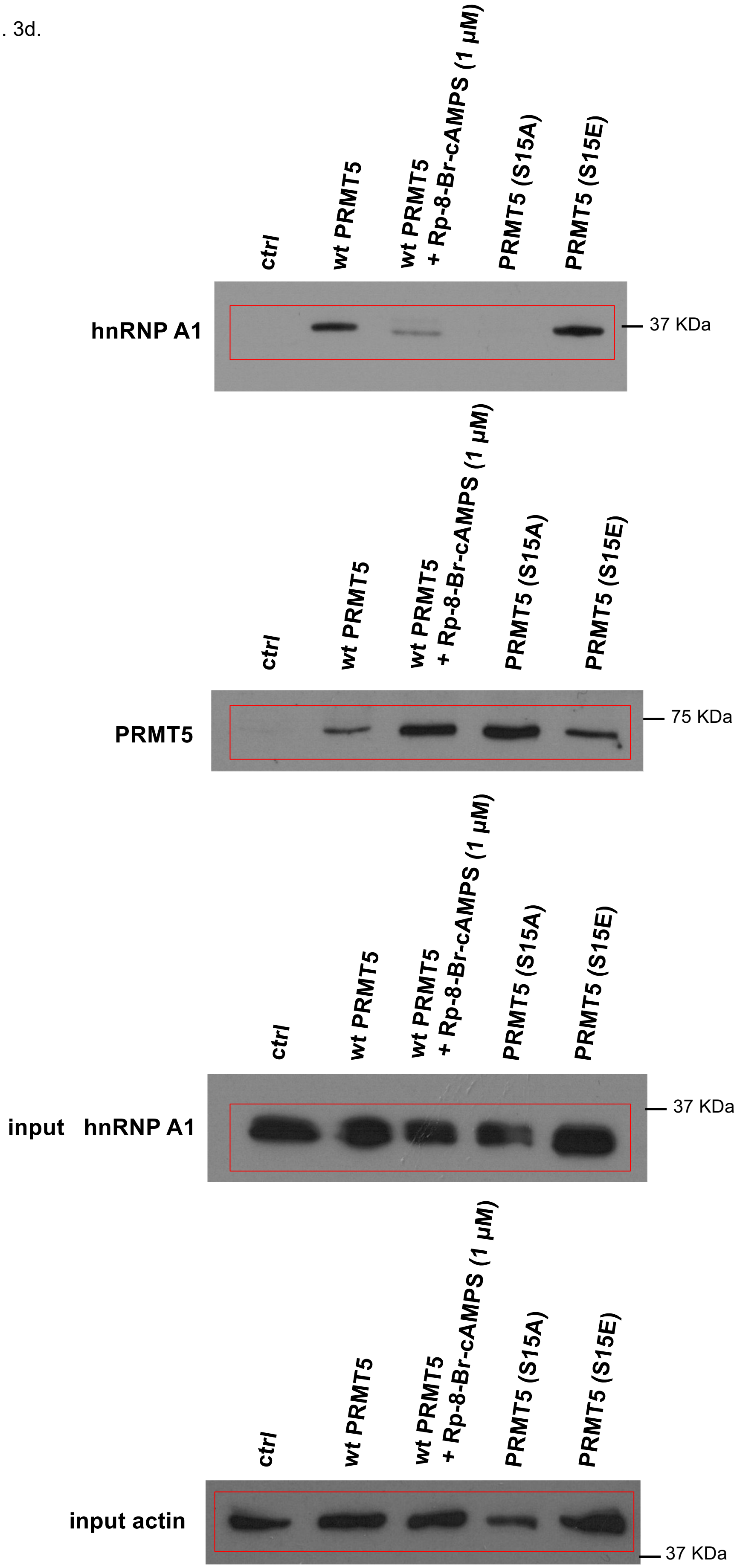
ctrl
wt PRMT5
wt PRMT5
+ Rp-8-Br-cAMPS (1 μM)
PRMT5 (S15A)
PRMT5 (S15E)
hnRNP A1
37 KDa
ctrl
wt PRMT5
wt PRMT5
+ Rp-8-Br-cAMPS (1 μM)
PRMT5 (S15A)
PRMT5 (S15E)
PRMT5
75 KDa
ctrl
wt PRMT5
wt PRMT5
+ Rp-8-Br-cAMPS (1 μM)
PRMT5 (S15A)
PRMT5 (S15E)
input hnRNP A1
37 KDa
ctrl
wt PRMT5
wt PRMT5
+ Rp-8-Br-cAMPS (1 μM)
PRMT5 (S15A)
PRMT5 (S15E)
input actin
37 KDa

Fig. 3e.

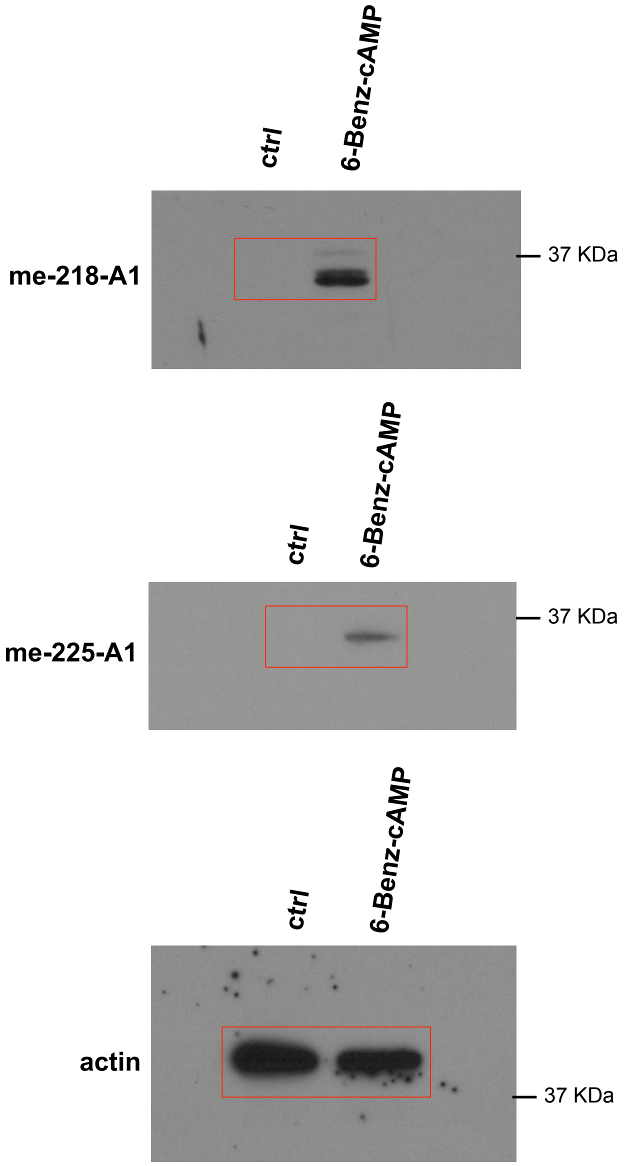
ctrl
6-Benz-cAMP
me-218-A1
37 KDa
ctrl
6-Benz-cAMP
me-225-A1
37 KDa
ctrl
6-Benz-cAMP
actin
37 KDa

Fig. 4f. *Top panel*.

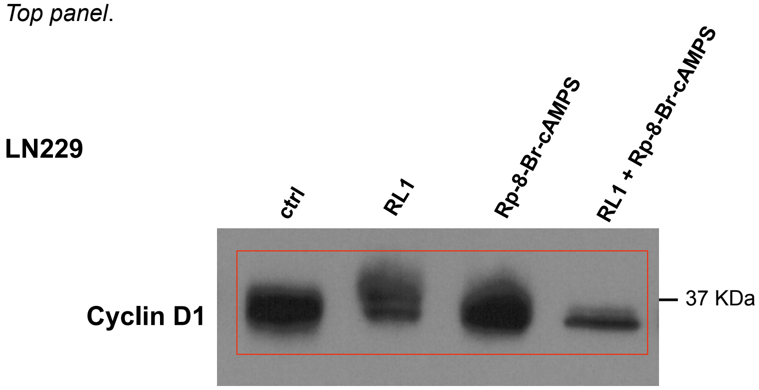

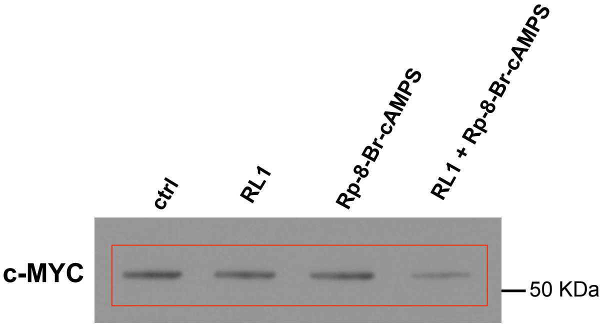

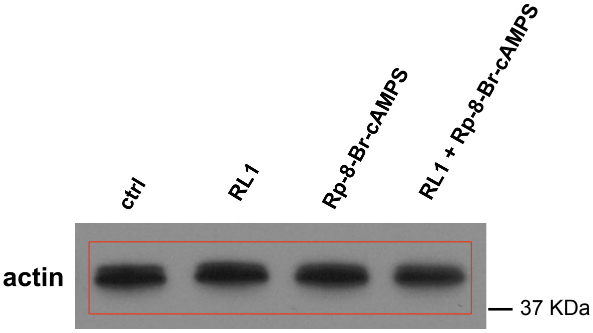

Fig. 4f. *Bottom panel*.

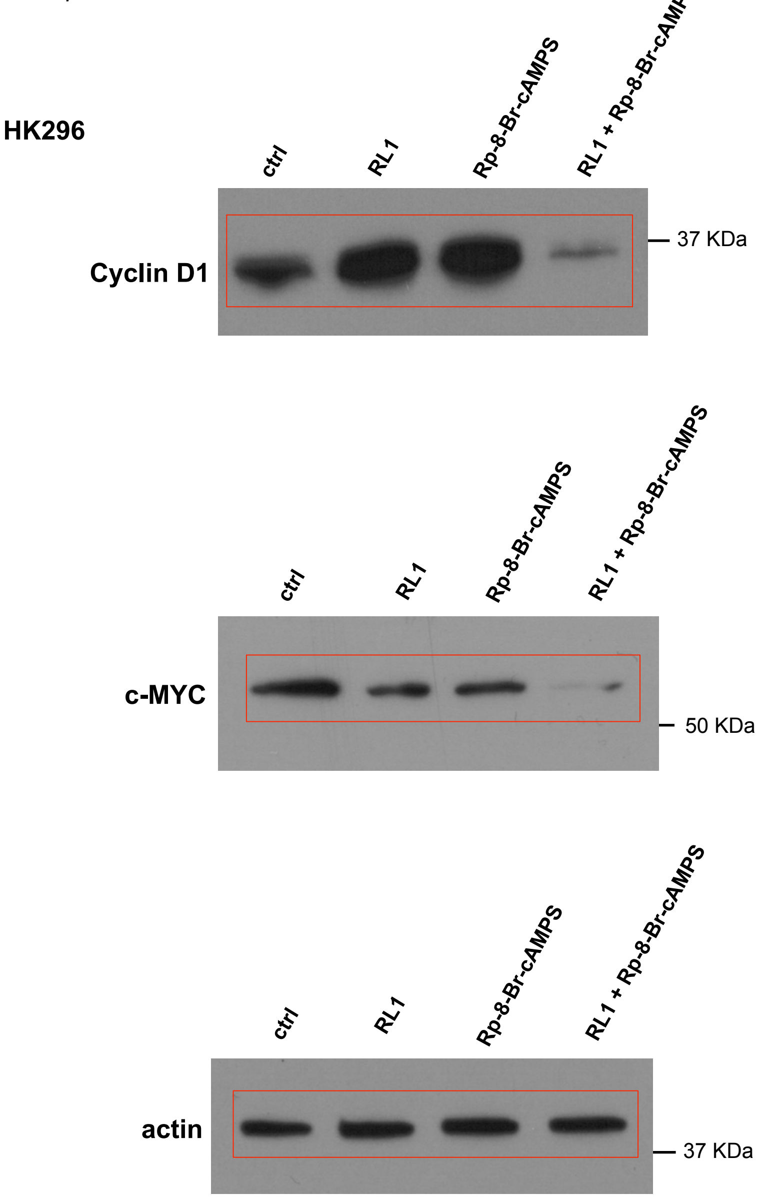

Supplementary Fig. S1a.

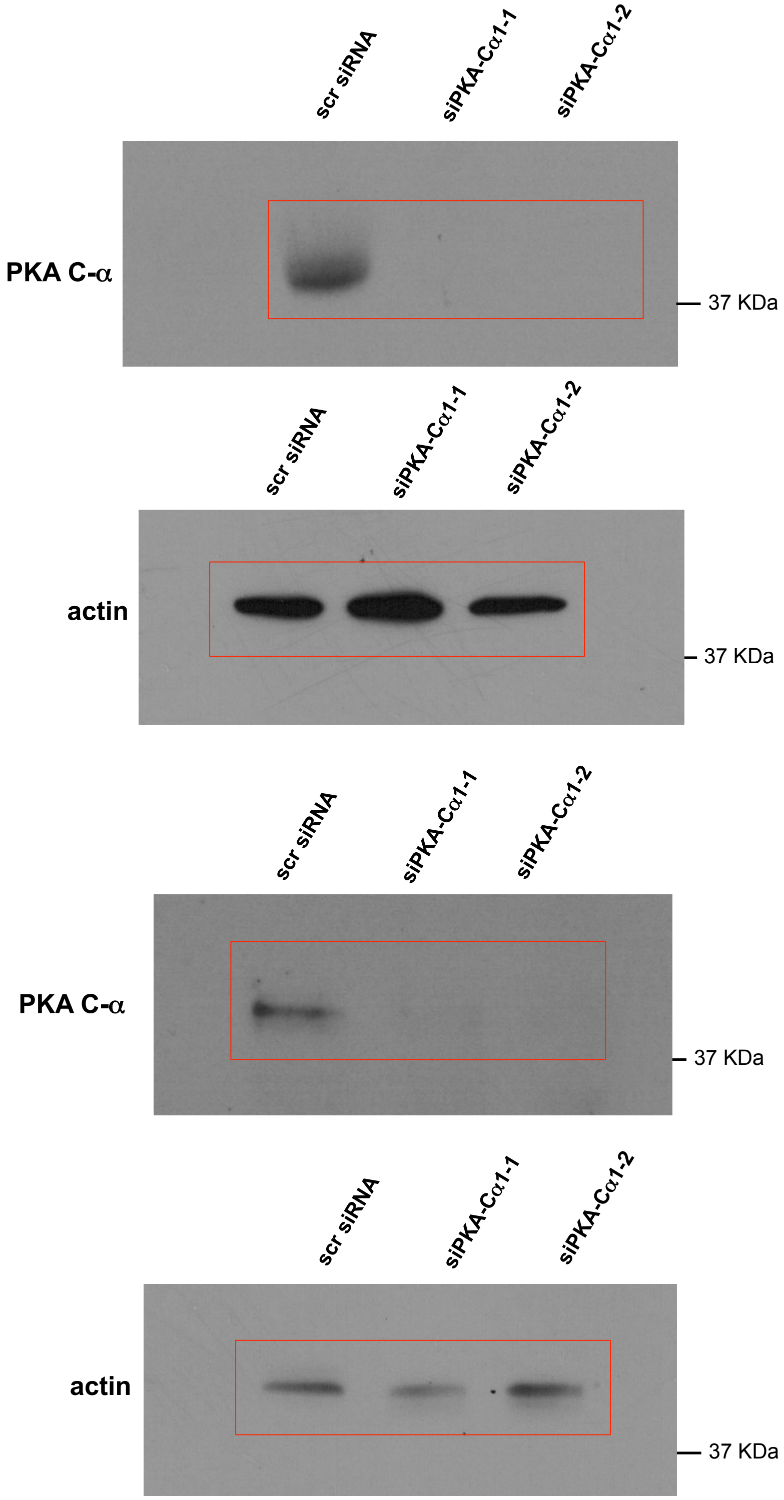

Supplementary Fig. S1b.

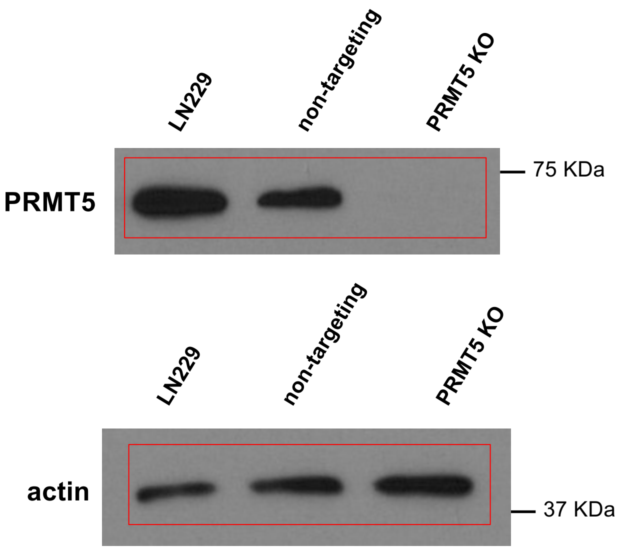

Supplement: Supplementary file 1 — Supplementary material 1 [file 11060_2026_5564_MOESM1_ESM.pdf]
