# Supplementary material for: PKA signaling modulates PRMT5/hnRNP A1-mediated IRES translation and dictates responses to mTOR inhibition in glioblastoma
Source: J Neurooncol. 2026 Jun 5;178(2):45. doi: 10.1007/s11060-026-05564-w (PMC13241469; doi:10.1007/s11060-026-05564-w)
Supplement: Supplementary file 2 — Supplementary material 2 [file 11060_2026_5564_MOESM2_ESM.pdf]

Online Resource 1. Supplemental Materials and Methods

PKA signaling modulates PRMT5/hnRNP A1-mediated IRES translation and dictates responses to mTOR inhibition in glioblastoma

Authors and Affiliations

Sunil Kumar [1], Angelica Benavides-Serrato [1], Jacquelyn T. Saunders [1], Robert N. Nishimura [1,3] and Joseph F. Gera [1,2,4,5]

[1]Department of Research & Development, Greater Los Angeles Veterans Affairs Healthcare System, Los Angeles, California, Department of [2]Medicine and [3]Neurology, David Geffen School of Medicine at UCLA, [4]Jonnson Comprehensive Cancer Center, [5]Molecular Biology Institute, University of California-Los Angeles, California

Corresponding Author: Joseph Gera, Ph.D., Greater Los Angeles VA Healthcare System, 16111 Plummer Street (151), Building 1, Room C111A, Los Angeles, CA 91343. Phone: (818) 895-9416; Fax: (818) 895-9554; E-Mail: jgera@mednet.ucla.edu

Cell Lines, Plasmids, Reagents, DNA transfections

LN229, A172, and M059J GBM lines were obtained from ATCC and short-term PDX HK296 cells were kindly provided by Dr. Harley Kornblum (Department of Molecular and Medical Pharmacology, UCLA). LN229-RFP-luc cells were from Cellomics. PRMT5-knockout LN229 cells were generated via CRISPR/Cas9-editing utilizing a human PRMT5 knockout kit as described by the manufacturer (Origene). Deletion mutants of PRMT5 and PKA-Cα were generated via PCR and cloned into pGBKT7 and pACT2, respectively. IRES reporter plasmids (pRCD1F, pRmycF) have been previously described [1]. Human PRMT5 cDNA cloned into pCMV3 harboring a C-terminal HA tag was from Sino Biological and site-directed mutagenesis was performed using the QuikChange II Site-Directed Mutagenesis kit (Agilent) to generate the nonphosphorylatable PRMT5 S15A or phosphomimetic PRMT5 S15E alleles using appropriate mutagenic primers. Active recombinant PKA-Cα was from R&D Systems. Antibodies to PKA-Cα, PRMT5, actin, hnRNP A1, cyclin D1, c-Myc, α-HA and control IgG were from Cell Signaling. α-PRMT5-FITC antibody was from Aviva Systems Biology and α-PKA-Cα-PE was from Abcam. Anti-methylation antibodies specific for me-R-218 and me-R-225 on hnRNP A1 were generated

in rabbits using the symmetric dimethylarginine peptides, R218 (GNDNFGR(Me2)GGNF) and R225 (GGNFSGR(Me2)GGFG) and subsequently affinity purified. For siRNA knockdowns, lines were transfected with 10 nmol/L siRNAs targeting human PKA-Cα (#1 & #2), or a non-targeting scrambled control sequence. ON-TARGETplus siRNAs were obtained from Horizon Discovery Biosciences and transfected using Lipofectamine RNAimax (ThermoFisher Scientific). 6-Bnz-cAMP, PP242, and Rapalink1 were purchased from APExBio and Rp-8-Br-cAMPS was from Cayman Chemical. DNA transfections were performed using Effectene transfection reagent according to the manufacturer's instructions (Qiagen).

Yeast two-hybrid screen

Standard yeast procedures were used to identify PRMT5 interacting protein partners utilizing yeast two-hybrid screens [2, 3]. Yeast two-hybrid screening was performed using the full-length human PRMT5 cDNA cloned into the yeast two-hybrid vector pGBKT7 in-frame with the Gal4 DNA-binding domain expressed in AH109 cells. Approximately 10^6 independent prey clones from cDNA libraries constructed in pACT2 from human brain (Takara Bio USA) and the GBM cell line LN229 were screened across three replicate experiments under low-stringency conditions to capture potential low-affinity interactors while minimizing false positives. Primary positives, including those encoding PKA-Cα (~7% of clones), were retested at high-stringency using selective media supplemented with 10–15 mM 3-amino-1,2,4-triazole to inhibit background growth. Dual reporter assays requiring activation of both *HIS3* and *LacZ* were employed to confirm interactions. Only clones demonstrating reporter activation dependent on both bait and prey plasmids, and loss of signal upon plasmid segregation or controls, were considered *bona-fide* interactors. Liquid β-galactosidase assays were conducted as previously described [4].

Colocalization, Immunohistochemistry, Coimmunoprecipitation, *in vitro* Kinase Assays, PKA activity assays, Western blot analyses

For immunofluorescence colocalization, cells were grown on coverslips and were fixed with 4% paraformaldehyde in PBS for 15 min at room temperature (or overnight at 4˚C) and washed three times for 5 min in 100 mM glycine containing PBS, followed by permeabilization with 0.1% Triton X-100 in PBS for 10 min. After blocking with 3% nonfat dry milk in PBS for 1 h, cells were incubated with FITC- and PE-labeled antibodies diluted in 1% BSA/PBS overnight at 4˚C. Cell nuclei were counterstained and mounted with a mounting medium with DAPI (Vectashield; Vector Laboratories). Immunofluorescence images were acquired via confocal microscopy (LSM 900, Zeiss). Protein co-localization was quantified using Pearson's correlation coefficient analysis within ImageJ. Pixel intensities from corresponding positions in two channels were paired to generate an intensity dataset. Pearson's values were interpreted as: +1, perfect positive correlation; > +0.5, positive association; 0, no correlation; −1, perfect negative correlation. Immunohistochemical quantification analyses were performed as previously described [5]. Quantification was conducted by investigators blinded to treatment allocation to minimize bias. Image acquisition was performed using identical microscope settings across all specimens to ensure comparability. Quantitative analysis was carried out using predefined thresholding parameters established based on negative control staining. For *in vitro* kinase assays, HA-immunoprecipitates from HA-PRMT5 transfected cells were incubated with active recombinant PKA-Cα and [γ-^{32}P]ATP at 30˚C for 25 min in a final volume of 15 µL of kinase buffer (100 mM Tris-HCL pH 7.5, 1 mM $MgCl_2$, 0.1 mg/ml BSA). Reactions were analyzed by SDS-PAGE and autoradiography. PKA activity assays were performed using the DetectX PKA activity kit as described by the manufacturer (Arbor Assays). Western blotting and coimmunoprecipitation experiments were performed as previously described [5].

IRES Reporter, Polysome analyses, Quantitative RT-PCR

For IRES reporter assays, reporters were transfected into cells and harvested 24 h following transfection and *Renilla* and firefly activities determined using the Dual-Glo Luciferase assay system (Promega). Polysome analyses were performed as previously described [6]. Briefly, cell extracts were

prepared and layered onto 15% to 50% sucrose gradients and spun at 38,000 rpm for 2 h at 4˚C in a SW40 Ti rotor (Beckman Coulter Life Sciences). Gradients were fractionated using a gradient fractionator system (BioComp Instruments) and the polysome profile of the gradients was monitored via absorbance at 260 nm. RNA was isolated and pooled into nonribosomal/monosomal and polysomal fractions. RNAs (100 ng) were subsequently used in quantitative reverse transcriptase-PCR analyses as described previously [6].

Cell Viability, Apoptosis, TUNEL assays

Cell viability was determined via Cell Titer-Glo luminescent cell assays (Promega) as described [7]. Cells were stained using a FITC-conjugated annexin V (Annexin V-FITC Early Apoptosis Detection kit, Cell Signaling Technology) to monitor apoptosis. Caspase-3 assays were performed using the Caspase-3 Cellular Assay Kit Plus from Enzo Life Sciences as described by the manufacturer. TUNEL staining of sections was performed utilizing the TACS-XL DAB In Situ Apoptosis Detection kit (Bio-Techne) according to the manufacturer's instructions. Combination index (CI) values were determined using CalcuSyn v2.0 software (Biosoft).

Orthotopic Xenograft Studies

All experiments were conducted in accordance with a protocol approved by the Institutional Animal Care and Use Committee of the Greater Los Angeles VA Healthcare System, following the guidelines set by the Association for the Assessment and Accreditation of Laboratory Animal Care. Intracranial xenografts were generated by implanting LN229-RFP-luc cells into 4- to 6-week-old female athymic NCr nude mice (Taconic), as previously described [7]. For bioluminescence imaging, mice received intraperitoneal injections of d-luciferin (Promega), and photon emissions were measured using an IVIS *in vivo* imaging system (PerkinElmer). Tumors were collected post-mortem for further immunohistochemical or polysome analysis.

Statistical Analyses

Statistical significance was determined by Student's *t* test or ANOVA followed by Tukey's post-hoc test. Overall survivals were calculated with the Kaplan–Meier method and compared using log-rank analysis. *P* values less then 0.05 were considered significant.

References for Supplemental Materials and Methods

1. Benavides-Serrato A, Saunders JT, Kumar S, Holmes B, Benavides KE, Bashir MT, Nishimura RN, Gera J (2023) m(6)A-modification of cyclin D1 and c-myc IRESs in glioblastoma controls ITAF activity and resistance to mTOR inhibition. Cancer letters 562: 216178 doi:10.1016/j.canlet.2023.216178
2. Sherman F (2002) Getting started with yeast. Methods in enzymology 350: 3-41 doi:10.1016/s0076-6879(02)50954-x
3. Gera JF, Hazbun TR, Fields S (2002) Array-based methods for identifying protein-protein and protein-nucleic acid interactions. Methods in enzymology 350: 499-512 doi:10.1016/s0076-6879(02)50981-2
4. Martin J, Masri J, Bernath A, Nishimura RN, Gera J (2008) Hsp70 associates with Rictor and is required for mTORC2 formation and activity. Biochemical and biophysical research communications 372: 578-583 doi:10.1016/j.bbrc.2008.05.086
5. Holmes B, Benavides-Serrato A, Saunders JT, Kumar S, Nishimura RN, Gera J (2021) mTORC2-mediated direct phosphorylation regulates YAP activity promoting glioblastoma growth and invasive characteristics. Neoplasia (New York, NY) 23: 951-965 doi:10.1016/j.neo.2021.07.005
6. Benavides-Serrato A, Saunders JT, Kumar S, Holmes B, Benavides KE, Bashir MT, Nishimura RN, Gera J (2023) m(6)A-modification of cyclin D1 and c-myc IRESs in glioblastoma controls ITAF activity and resistance to mTOR inhibition. Cancer letters 562: 216178 doi:10.1016/j.canlet.2023.216178
7. Saunders JT, Kumar S, Benavides-Serrato A, Holmes B, Benavides KE, Bashir MT, Nishimura RN, Gera J (2023) Translation of circHGF RNA encodes an HGF protein variant promoting glioblastoma growth through stimulation of c-MET. Journal of neuro-oncology 163: 207-218 doi:10.1007/s11060-023-04331-5

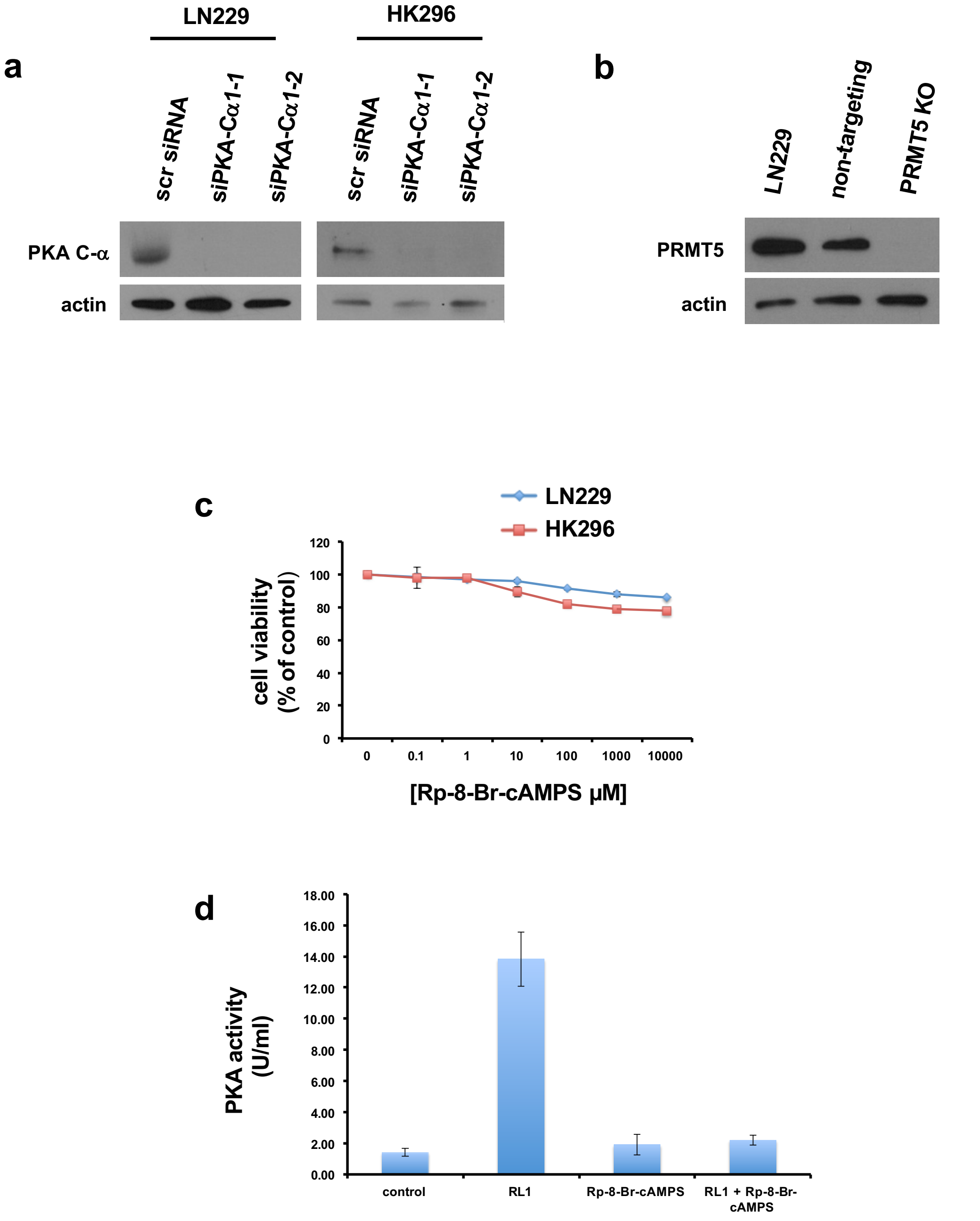

Supplementary Fig. S1. a Knockdown of PKA-Cα expression via two independent targeting siRNAs (siPKA-Cα1-1, siPKA-Cα1-2) in LN229 and HK296 cells. Cells were treated with control, non-targeting scrambled siRNA (scr siRNA), siPKA-Cα1-1, and siPKA-Cα1-2, and lysates immunoblotted for the indicated proteins. **b** CRISPR/Cas9 knockout of PRMT5 in LN229 cells. **c** Cell viability of LN229 and HK296 cells treated with the indicated concentrations of Rp-8-Br-cAMPS at 48 h. **d** PKA activity in harvested intracranial xenografted cells from the indicated treatment groups at day 32 post-implantation.

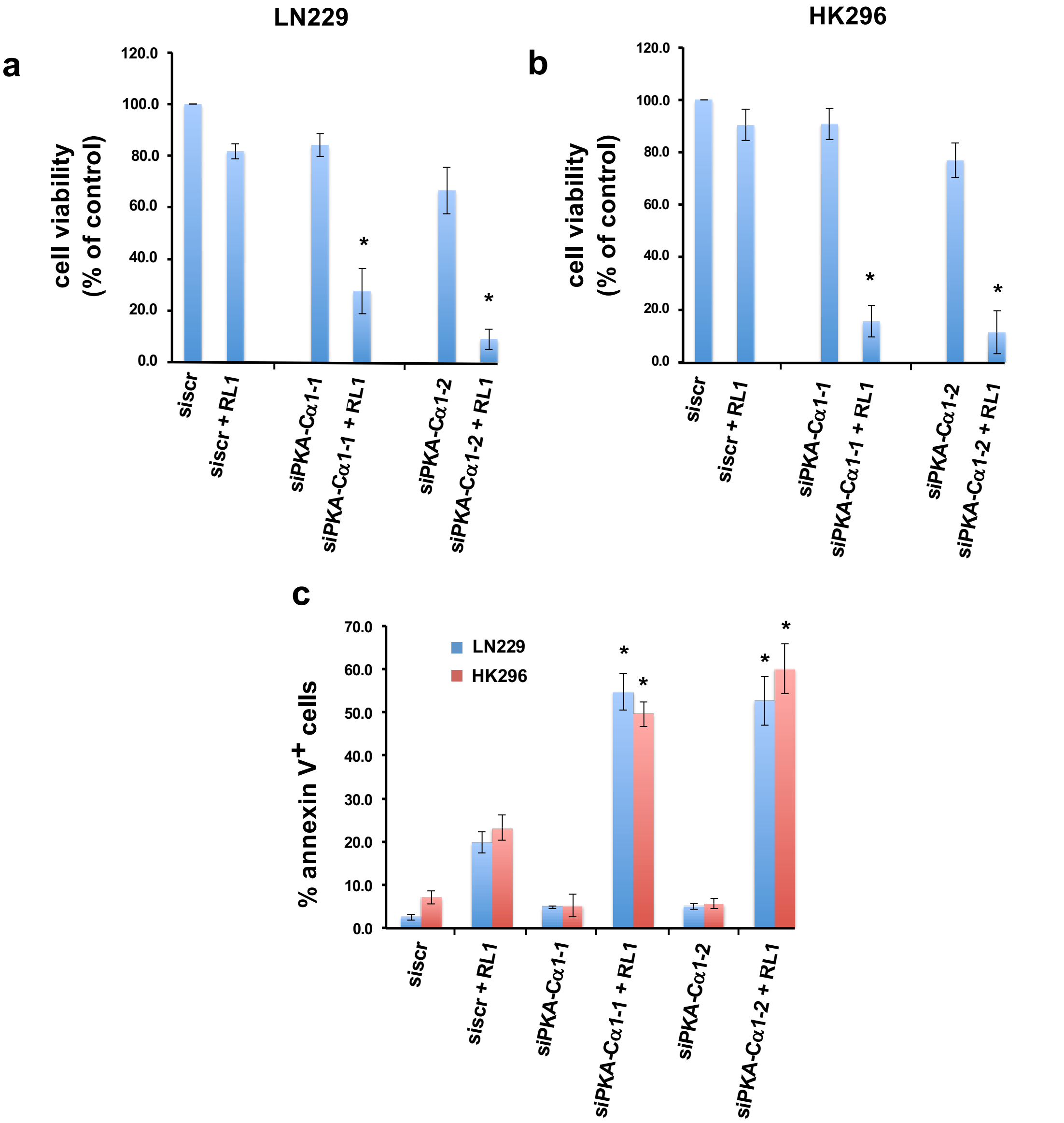

Supplementary Fig. S2. a Cell viability of LN229 cells treated with non-targeting control scrambled siRNA (siscr) or PKA-Cα-targeting siRNAs (siPKA-Cα1-1 or siPKA-Cα1-2) in the absence or presence of RL1 (1 nM, 48 h) as indicated. Mean ± S.D., n = 3. *, *P* < 0.05 (siPKA-Cα1-1 + RL1 or siPKA-Cα1-2 + RL1 versus siPKA-Cα1-1 or siPKA-Cα1-2, respectively). **b** As in **a**, except in GBM PDX HK296 cells. **c** Apoptosis induction following treatments with the indicated siRNAs in the absence or presence of RL1 (1 nM, 48 h) was assessed via annexin V-FITC staining in LN229 and HK296 cells. Mean ± S.D., n = 3. *, *P* < 0.05 (siPKA-Cα1-1 + RL1 versus siPKA-Cα1-1 or siPKA-Cα1-2 + RL1 versus siPKA-Cα1-2 in LN229 and HK296 cells).

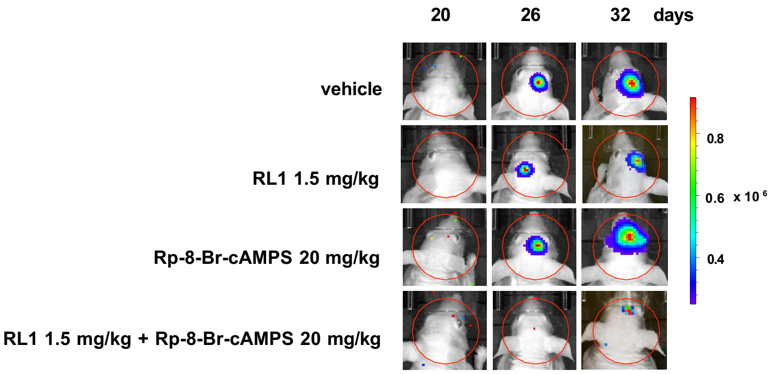

Supplementary Fig. S3. Efficacy of combination RL1 and Rp-8-Br-cAMPS treatment in orthotopic LN229-luc-tagged tumors. Representative BLI obtained at days 20, 26 and 32 as described in Fig. 5a.
